# Parallel Structural Evolution of Mitochondrial Ribosomes and OXPHOS Complexes

**DOI:** 10.1093/gbe/evv061

**Published:** 2015-04-09

**Authors:** Eli O. van der Sluis, Heike Bauerschmitt, Thomas Becker, Thorsten Mielke, Jens Frauenfeld, Otto Berninghausen, Walter Neupert, Johannes M. Herrmann, Roland Beckmann

**Affiliations:** ^1^Gene Center and Center for integrated Protein Science Munich (CiPSM), Department of Biochemistry, University of Munich, Germany; ^2^Cell Biology, University of Kaiserslautern, Germany; ^3^Max Planck Institute for Molecular Genetics, UltraStrukturNetzwerk, Berlin, Germany; ^4^Institut für Medizinische Physik und Biophysik, Charité, Berlin, Germany; ^5^Max Planck Institute of Biochemistry, Martinsried, Germany; ^6^Present address: Department of Medical Biochemistry and Biophysics, Karolinska Institutet, Stockholm, Sweden

**Keywords:** ribosome, nonadaptive evolution, mitochondrial evolution, cryo-electron microscopy

## Abstract

The five macromolecular complexes that jointly mediate oxidative phosphorylation (OXPHOS) in mitochondria consist of many more subunits than those of bacteria, yet, it remains unclear by which evolutionary mechanism(s) these novel subunits were recruited. Even less well understood is the structural evolution of mitochondrial ribosomes (mitoribosomes): while it was long thought that their exceptionally high protein content would physically compensate for their uniquely low amount of ribosomal RNA (rRNA), this hypothesis has been refuted by structural studies. Here, we present a cryo-electron microscopy structure of the 73S mitoribosome from *Neurospora crassa*, together with genomic and proteomic analyses of mitoribosome composition across the eukaryotic domain. Surprisingly, our findings reveal that both structurally and compositionally, mitoribosomes have evolved very similarly to mitochondrial OXPHOS complexes via two distinct phases: A constructive phase that mainly acted early in eukaryote evolution, resulting in the recruitment of altogether approximately 75 novel subunits, and a reductive phase that acted during metazoan evolution, resulting in gradual length-reduction of mitochondrially encoded rRNAs and OXPHOS proteins. Both phases can be well explained by the accumulation of (slightly) deleterious mutations and deletions, respectively, in mitochondrially encoded rRNAs and OXPHOS proteins. We argue that the main role of the newly recruited (nuclear encoded) ribosomal- and OXPHOS proteins is to provide structural compensation to the mutationally destabilized mitochondrially encoded components. While the newly recruited proteins probably provide a selective advantage owing to their compensatory nature, and while their presence may have opened evolutionary pathways toward novel mitochondrion-specific functions, we emphasize that the initial events that resulted in their recruitment was nonadaptive in nature. Our framework is supported by population genetic studies, and it can explain the complete structural evolution of mitochondrial ribosomes and OXPHOS complexes, as well as many observed functions of individual proteins.

## Introduction

The 3D structure of ribosomes has been investigated extensively by both cryo-electron microscopy (cryo-EM) and X-ray crystallography: It comprises a core region that is universally conserved, and a peripheral shell region that varies between the three domains of life (Melnikov et al. 2012). Among cytoplasmic ribosomes, most of the structural variation is found within the eukaryotic domain of life, where the peripheral shells consist of numerous highly variable rRNA expansion segments and several eukaryote-specific ribosomal proteins. The structural variation between and within bacterial and archaeal ribosomes is largely restricted to a few ribosomal proteins, and is comparatively small.

The structure of organellar ribosomes on the other hand remains much poorer characterized than that of cytoplasmic ribosomes. Mitochondria as well as chloroplasts have inherited a highly reduced genome from their once free-living bacterial ancestor that harbors genes involved in photosynthesis (chloroplasts), oxidative phosphorylation (OXPHOS, mitochondria), and protein synthesis (both). Yet, although the antibiotic sensitivity spectrum of both organellar ribosomes is very similar to that of bacteria, on a structural level only chloroplast ribosomes closely resemble bacterial ones: rRNA sequences and secondary structures are highly similar, ribosomal proteins are well-conserved, and a mere six “plastid-specific ribosomal proteins” have been recruited (Sharma et al. 2007).

Mitochondrial ribosomes (mitoribosomes) on the other hand, as exemplified by the well-characterized mammalian 55S mitoribosome, have undergone two distinct modes of molecular evolution: Their rRNA fraction has evolved “reductively” down to approximately half the number of nucleotides that is commonly found in bacteria, and its protein fraction has evolved “constructively” to approximately double the mass in bacteria (Sharma et al. 2003). The latter is due to 1) length increase of ribosomal proteins from bacterial descent (B-RPs) and 2) the recruitment of approximately 25 “mitochondrion-specific ribosomal proteins” (MS-RPs; Smits et al. 2007; Desmond et al. 2011). This concomitant rRNA-decrease and r-protein increase has led to the widely spread hypothesis that MS-PRs may physically replace missing rRNA segments by acting as “molecular prostheses” (O'Brien 2002). This prosthetic hypothesis has been refuted, however, by the cryo-EM structure of the 55S mitoribosome, which revealed that MS-RPs mostly occupy novel peripheral positions and that many voids that result from rRNA-reduction remain unfilled (Sharma et al. 2003). Very recently, higher resolution cryo-EM structures of the large (39S) and the small (28S) subunit of the 55S confirmed these findings, and provided a wealth of detailed structural insights into the 55S architecture (Amunts et al. 2014; Kaushal et al. 2014). Briefly, unlike that of any other ribosome, the core of the 55S mitoribosome is highly porous, while the surrounding proteinaceous shell has grown much more complex than that of the ancestral bacterial ribosome.

Following refutation of the prosthetic hypothesis, mitochondrion-specific functional roles have been attributed to MS-RPs, based on their location in the 3D structure. For example, MS-RPs in the vicinity of the mRNA entry channel on the small subunit (SSU) were proposed to be involved in the regulation of translation initiation, and those in vicinity of ribosomal exit tunnel on the large subunit (LSU) were proposed to be involved in membrane binding or release of nascent membrane proteins (Sharma et al. 2003; Amunts et al. 2014), because many mitochondrial genomes exclusively encode integral membrane proteins. Although at first sight such reasoning may seem plausible, it does not take the evolutionary origin of MS-RPs into account and thus it may represent an incomplete view in which other functions are overlooked. First, physical proximity to a certain functional site on the ribosome does not necessarily imply that an MS-RP is actually involved in this function. And second, even if a certain MS-RP is currently involved in a mitochondrion-specific function, this does not imply that it initially evolved as an adaptation to a related mitochondrion-specific demand: The MS-RP may have been recruited for other reasons, and acquired the mitochondrion-specific function only secondarily. The pitfall of “adaptive storytelling” in evolutionary biology is long known (Gould and Lewontin 1979), and hence it is often advocated that nonadaptive recruitment mechanisms should be considered first (Stoltzfus 1999; Lynch 2007a, 2007b; Gray et al. 2010). The latter are based on the neutral theory (Kimura 1983), which generally serves as a null hypothesis in molecular evolution.

In order to shed more light on the structural evolution of mitoribosomes and to better understand the present function of MS-RPs, we have determined the cryo-EM structure of a mitoribosome with a nonreduced rRNA content. We interpret this structure in an evolutionary perspective together with mitochondrial OXPHOS complexes, which are structurally and functionally much better characterized than mitoribosomes, but whose evolutionary origins are only partially understood. This perspective reveals two common principles in the structural evolution of mitochondrial ribosomes and OXPHOS complexes, which we argue resulted from two common evolutionary mechanisms acting exclusively on mitochondrially encoded components. From this combination of common principles, we then infer a common structural function for the altogether approximately 75 MS-RPs and OXPHOS proteins, which is neither related to OXPHOS, nor to translation. In other words, by resolving the evolutionary history of mitochondrial ribosomes and OXPHOS complexes, we provide a simple framework that sheds a new light onto the dramatically increased complexity of the ancient functional core of mitochondria.

## Materials and Methods

### Mitoribosome Isolation

Mitoribosomes were isolated from *Neurospora crassa* strain K5–15-23–1 (Nargang et al. 2002), essentially as described previously (Neupert et al. 1979). All steps were performed at 4 °C. Briefly, approximately 2 g of total mitochondrial protein in TAM buffer (30 mM Tris-HCl pH 7.5; 100 mM NH_4_Cl; 10 mM MgCl_2_) was isolated by differential centrifugation from approximately 0.5 kg of mycelium. Mitochondria were lysed by the addition of 2% (w/v) Triton-X100 and crude ribosomes were pelleted (120 min ∼185,000 × g) through a 1.4 M sucrose cushion in TAM-MDC buffer (= TAM supplemented with 4 mM 2-mercaptoethanol, 0.05% n-Dodecyl β-D-maltoside (DDM) and 0.0075% cardiolipin). Ribosomal pellets were resuspended in TAM-MDC, and 73S peaks were isolated from a 10–30% (w/v) sucrose gradient in the same buffer after 10 h centrifugation at 85,000 × g. No material was frozen until vitrification of the ribosomes on carbon coated holey cryogrids. Contaminations of cytoplasmic 80S ribosomes could not be detected by mass spectrometric analyses.

### Cryo-EM and Image Processing

Cryo-EM data were collected on a Tecnai G2 Polara TEM (FEI) operated at 300 kV, and micrographs were scanned on a Heidelberg drum scanner with a pixel size of 1.23 Å on the object scale. CTF correction by CTFFIND (Mindell and Grigorieff 2003), automated particle picking by SIGNATURE (Chen and Grigorieff 2007), and image processing by SPIDER (Frank et al. 1996) were performed essentially as described (Seidelt et al. 2009). An empty *Escherichia coli* 70S ribosome structure filtered from approximately 25 to 30 Å was used as initial reference. Two data sets were refined jointly: 324,801 particles of 73S ribosomes complexed with *N. crassa* Oxa1 (van der Sluis EO, et al. unpublished data) and 267,830 particles of vacant 73S ribosomes, 208,737 of which were used in the final back-projection for preparation of the figures, following elimination of 59,093 particles based with low cross-correlation coefficients. The resolution of the final structure as estimated from the Fourier Shell Correlation was approximately 7.5 Å based on the 0.5 σ criterion. Atomic models of *E. coli* ribosomal subunits (3FIH, 3FIK) were docked as three rigid bodies (SSU head separately from the SSU body) into the experimental electron density map using UCSF Chimera (Goddard et al. 2007), mitochondrion-specific density was subsequently separated into rRNA and protein by visual inspection.

### Structural Analysis of OXPHOS Complexes

The membrane region of *Thermus thermophilus* complex I (Efremov et al. 2010) was docked as four separate rigid bodies into the electron density map of *Yarrowia lipolytica* complex I (Hunte et al. 2010) and mitochondrion-specific electron density was isolated with aid of the color zone function in UCSF Chimera (Goddard et al. 2007). Atomic models of complexes II–V were subdivided into MS- and core proteins, and colored accordingly after filtering to 7 Å resolution to facilitate comparison with the mitoribosome structure. The Research Collaboratory for Structural Bioinformatics (RCSB) accession numbers of the atomic models are: complex I: 3M9S; complex II: 2FBW; complex III: 1BGY (subunit 11) and 1PPJ (all other subunits); complex IV: 1V54; and complex V: 2WSS (F_1_-part) and 2XND (F_O_-part). The positions of bound cytochrome c on complexes III and IV were obtained from 3CX5 and 1ZYY, respectively.

### Mitochondrial Gene Length Analysis

Gene lengths were manually downloaded from completely sequenced mitochondrial genomes available from NCBI, with the exception of the *N. crassa* genome obtained from the Broad Institute. With the exclusion of parasitic organisms, most of the available plant, algal, protist, fungal, cnidarian, poriferan, and placozoan genomes were included in the analysis; the bilaterian phylogenetic tree on the other hand was broadly sampled in order to avoid bias against overrepresented taxa such as vertebrates. Total rRNA lengths were obtained by summing the annotated lengths of (fragmented) SSU, LSU and, when present, 5S rRNA sequences, with the exclusion of gene duplicates. The indicated tRNA lengths are averages of all annotated tRNA genes present in a mitochondrial genome. The total length of mitochondrially encoded OXPHOS complexes is the sum of all the amino acid residues encoded by the genes *nad1*, *nad2*, *nad3*, *nad4*, *nad4L*, *nad5*, and *nad6* genes (complex I); *cytb* (complex III); *cox1*, *cox2*, and *cox3* (complex IV); and *atp6* and *atp8* (complex V). Mitochondrial genomes lacking one or more of the latter, as well as gene duplicates were excluded from the analysis.

### Analyses of rRNA Base Pairing

The comparative RNA website (Cannone et al. 2002) hosts base pairing conservation values that were derived from an alignment of 326 bacterial, 312 mitochondrial, and 103 plastid rRNAs (Mears et al. 2002). From this website, we extracted the “WCGU” values, that is, the number of sequences in this alignment having a canonical base pair (Watson:Crick or G:U) at a given interaction site, divided by the total number of sequences in the alignment with 2 nt at that site. Note that this calculation is not influenced by rRNA length reduction, because interaction sites that lack one or two bases (i.e., gaps in the alignment) are not included. We subdivided the total number of interaction sites into four groups: “regular” secondary sites, secondary sites involved in an A-minor motif (Nissen et al. 2001; Bokov and Steinberg 2009), canonical tertiary sites, and noncanonical tertiary sites. The conservation values of the latter were obtained by subtracting the canonical conservation values from 100%. For visualization, the resulting 12 individual data sets were numerically ordered according to their conservation value and plotted against the number of sites in each group.

### Calculation of Structural Deficiency

The structural deficiencies (Fernandez and Lynch 2011) were calculated from crystal structures of the (combined) mitochondrially encoded proteins or their counterparts, using the default settings of the desolvation plugin of YAPview (available from http://sourceforge.net/projects/protlib/files/ yapview/, last accessed April 2015). Averages and standard deviations were calculated when crystal structures from more than one organism were available. The following subunits and PDB files were used: All four subunits from complex II: 1NEK (bacterial) and 1ZOY (mitochondrial); cytochrome *b* from complex III: 1ZRT and 2FYN (bacterial), 1EZV, 1BCC, 1BGY, 1NTM (mitochondrial); cytochrome *f*, cytochrome *b*_6_, subunit IV, PetG, and PetL from cytochrome *b*_6_*f*: 1VF5 (bacterial), 1Q90 (chloroplast); and Cox1, Cox2, and Cox3 from complex IV: 1M57 and 1QLE (bacterial), 1V54 and 1OCC (mitochondrial).

## Results

### Mitoribosome Composition in Model Organisms

**Fig. 1.—:**
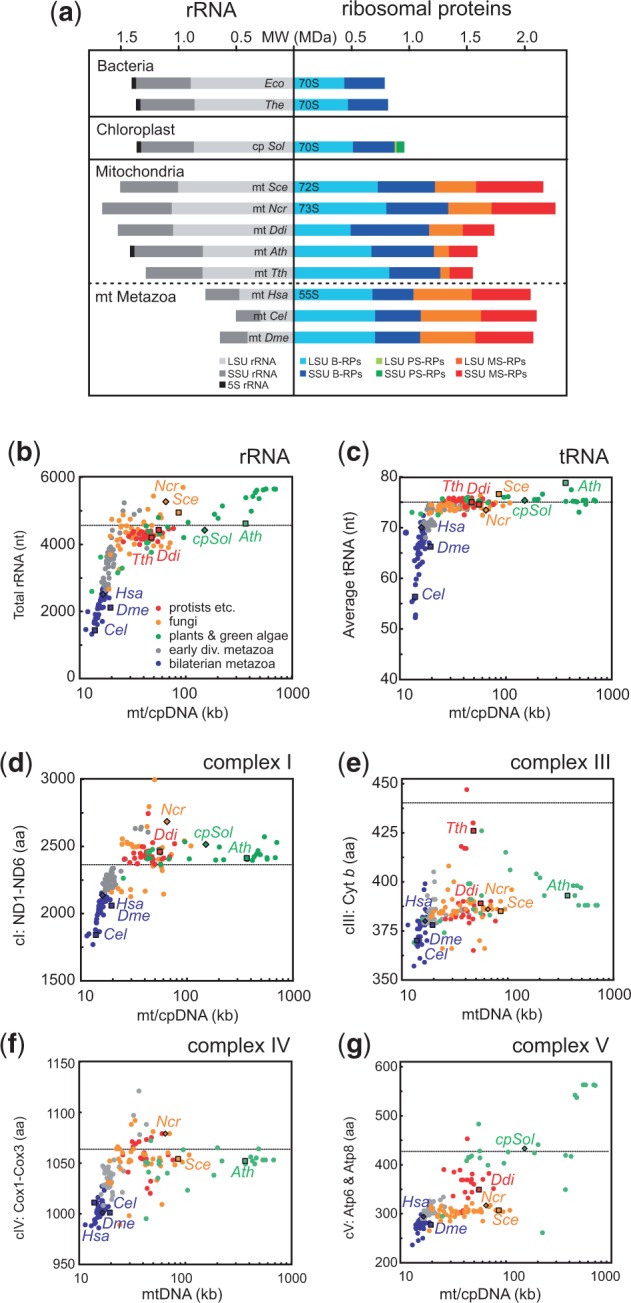
Ribosome composition in model organisms and mitochondrial gene length analysis. (*a*) An elevated protein content is a general property of mitochondrial ribosomes, but a reduced rRNA content is not. The predicted masses (in MDa) of rRNA (left) and proteins (right) of bacterial and organellar ribosomes from 11 model organisms is shown, with experimentally determined sedimentation coefficients indicated. Metazoa are depicted below the dashed line. PS-RPs, plastid-specific ribosomal proteins; mt, mitochondrion; cp, chloroplast; *Eco*, *Escherichia coli*; *The*, *Thermus thermophilus*; *Sol*, *Spinacea oleracea*; *Sce*, *Saccharomyces cerevisiae*; *Ncr*, *Neurospora crassa*; *Ddi*, *Dictyostelium discoideum*; *Ath*, *Arabidopsis thaliana*; *Tth*, *Tetrahymena thermophila*; *Hsa*, *Homo sapiens*; *Cel*, *Caenorhabditis elegans*; *Dme*, *Drosophila melanogaster.* (*b***–***g*) Length reduction is a unique property of metazoan mitochondrial genes. Total rRNA lengths (*b*, in nucleotides), average tRNA lengths (*c*, in nucleotides), and the (summed) lengths of the mitochondrially encoded subunits (in amino acid residues) of complex I (ND1-ND6: *d*), complex III (Cyt *b*: *e*), complex IV (Cox1-Cox3: *f*), and complex V (Atp6 and Atp8: *g*) are plotted as a function of the total organellar genome size (in kilo base pairs: kb). Dotted lines indicate the approximate values in bacteria, all values are provided in supplementary table SI, Supplementary Material online.

When studying the structural evolution of the mitoribosome it is apparent that the mitoribosomal proteome is in general much larger than in bacteria. Unlike the mitoribosomal proteome, however, the mitoribosomal RNA content of model organisms was thus far not systematically analyzed. Hence, from nine eukaryotic model organisms, we first related the mitoribosomal rRNA content (deduced from completely sequenced mitochondrial genomes) to their r-protein content that was previously predicted by comparative genomics (fig. 1*a*; Smits et al. 2007). The total mitochondrial ribosomal protein (MRP) mass of each organism ranges from approximately 1.3 to 2.3 MDa, and hence all analyzed mitoribosomes can be classified as “protein-rich,” relative to the approximately 0.8 MDa total r-protein mass of the bacterial reference organisms *E. coli* and *T. thermophilus*. Cleavable mitochondrial targeting sequences (MTSs) are responsible for only a small part of this length-increase of at least 0.5 MDa: The average length of the 67 known MTSs of yeast MRPs is approximately 25 residues (∼3 kDa; Vogtle et al. 2009), and assuming that this value is not significantly different for the remaining proteins, the estimated MTS mass of the yeast mitoribosome is merely approximately 10% of its total MRP mass. Furthermore, among endosymbiotic organelles the dramatic mass increase of r-protein is unique for the mitoribosome, as the chloroplast ribosome, exemplified here by *Spinacea oleracea*, gained altogether only approximately 170 kDa (fig. 1*a*; data from Yamaguchi and Subramanian [2000] and Yamaguchi et al. [2000]). As noted previously (Smits et al. 2007; Desmond et al. 2011), the recruitment of most MS-RPs occurred very early in eukaryote evolution, that is, before divergence of the major eukaryotic lineages, whereas lineage-specific MS-RPs were recruited afterward. However, the evolutionary mechanisms that underlay MS-RP recruitment have thus far remained unclear.

In contrast to the fairly uniform gain of protein mass, the total mitoribosomal RNA mass ranges from approximately 0.5 MDa in *Caenorhabditis elegans* to approximately 1.6 MDa in *N. crassa* (fig. 1*a*), corresponding to a loss of 0.9 MDa or a gain of 0.2 MDa, respectively, relative to bacteria. Importantly, there is no correlation between the loss of rRNA and the gain of r-protein. Thus, the analysis of this set of model organisms indicates that elevated protein content is a general property of mitoribosomes, whereas a reduction of rRNA content is not.

### Reductive Evolution of rRNAs in Metazoa

We investigated the apparent peculiarity of rRNA reduction more broadly, by analyzing a large and phylogenetically balanced set of greater than 200 mitochondrial genomes covering the entire eukaryotic domain (supplementary table S1, Supplementary Material online). Because it has been shown that rRNA lengths can correlate with mitochondrial genome size (Andersson and Kurland 1991; Schneider and Ebert 2004), we plotted the rRNA length of each organism against the size of its mitochondrial genome. The distribution of the total number of nucleotides encoding rRNA shows a continuum ranging from approximately 1,500 to 5,500, that is unevenly occupied by different taxonomic groups (fig. 1*b*). Length-reduced rRNA appears to be a derived, primarily metazoan characteristic: Essentially all other taxonomic groups contain roughly the same amount of rRNA as the ancestral bacterial ribosome.

The most dramatically reduced rRNAs are found among the bilaterian metazoa, a large and diverse taxonomic group of animals with bilateral symmetry, which includes mammals. Within the latter, there is remarkably little variation. And finally, early diverging metazoa (porifera, cnidaria, and placozoa) exhibit intermediate rRNA lengths that bridge the greater than 1 kb gap between the bilateria and nonmetazoan eukaryotes (fig. 1*b*).

Taking into account that metazoa appeared long after the first eukaryotes, the combined analysis of MRP- and rRNA data indicates that the constructive phase of mitoribosomal protein (M-RP)-gain largely occurred before the reductive phase of rRNA-loss: The (mito)ribosome first acquired numerous MS-RPs early in eukaryote evolution, converting it from “rRNA-rich but protein-poor” (as in bacteria) to “rRNA-rich and protein-rich.” Plants, fungi, protests, and most algae have retained such rRNA-rich mitoribosomes. Only much later, that is, early in metazoan evolution, the reduction of rRNA was initiated that resulted in protein-rich mitoribosomes with varying degrees of rRNA poverty. We, thus, conclude that 1) the elevated protein content of mitoribosomes did not evolve in response to rRNA reduction, but preceded it. And 2) mitoribosomes constitute a highly heterogeneous class of exceptionally protein-rich ribosomes with dramatically varying amounts of rRNA. And thus, the well-characterized mammalian “rRNA-poor and protein-rich” 55S mitoribosome is not representative for eukaryotes, but rather exceptional.

### Parallel Reductive Evolution of Mitochondrial rRNAs and OXPHOS Genes in Metazoa

In order to investigate whether the reductive evolution of mitochondrial rRNAs is an exceptional or a more general metazoan phenomenon, we also analyzed the lengths of mitochondrially encoded tRNAs (fig. 1*c*) and OXPHOS proteins from complexes I, III, IV, and V (fig. 1*d*–*g*). Indeed, all classes of mitochondrial genes display length reduction and the largest reduction is observed among the bilaterian metazoa and, similar to the rRNAs, early diverging metazoa exhibit intermediate lengths. While rRNAs can be reduced in length down to only one-third of their ancestral length, the length reductions observed for OXPHOS proteins may be less dramatic for several possible reasons: 1) Random deletions in OXPHOS genes, but not in rRNAs, must be multiples of 3 nt in order to maintain reading frame; 2) rRNAs, but not OXPHOS complexes, are thought to have an early evolutionary history of gradual growth (Bokov and Steinberg 2009), which may provide an intrinsic “buffer” toward deletions; 3) for OXPHOS complexes, a high porosity analogous to the mammalian mitoribosome may be structurally more challenging because impermeability of the mitochondrial inner membrane must be maintained. However, even the truncation of three transmembrane segments from the ND2 subunit of complex I can apparently be tolerated in *C. elegans* and *Bos taurus* (Birrell and Hirst 2010). In any case, our data indicate that mitochondrial gene length reduction has been a general and gradual ratchet-like process that was initiated early in metazoan evolution. And importantly, this implies that the explanation(s) for the porous nature of the mammalian mitoribosome must be sought in the process(-es) underlying the deletion bias of metazoan mitochondrial genomes, and not in unique functional properties of mitochondrial translation systems.

### Parallel Constructive Evolution of Mitochondrial Ribosomes and OXPHOS Complexes

We noted that OXPHOS complexes I (Gabaldon et al. 2005; Cardol 2011), III and IV (see supplementary results and tables S2–S4, Supplementary Material online, and [Gawryluk et al. 2012]), and V (Lapaille et al. 2010) have undergone an early phase of constructive evolution that is very similar to that observed for the mitoribosome, at least on a compositional level: Early in eukaryote evolution they recruited a large number of mitochondrion-specific OXPHOS proteins (MS-OPs), commonly referred to as “supernumerary” or “accessory” proteins. Similarly to the MS-RPs of the mitoribosome (Smits et al. 2007), most of these MS-OPs are ubiquitous among eukaryotes, whereas some are lineage specific. MS-OPs are generally involved in the stabilization, regulation, or folding/assembly of these OXPHOS complexes (Berry 2003; Zickermann et al. 2010); neither of them is directly involved in catalysis. Hence, these four OXPHOS complexes have evolved qualitatively similarly to the mitoribosome, that is, all underwent a constructive phase early in eukaryote evolution and a reductive phase during metazoan evolution.

On a structural level, the constructive evolution of mitochondrial OXPHOS complexes can be visualized by highlighting MS-OPs in available crystal structures. Such an analysis reveals that the conserved core of bacterial and eukaryotic OXPHOS complexes has remained essentially unaltered, whereas the MS-OPs of eukaryotes are distributed peripherally around this core as an additional shell (supplementary fig. S1, Supplementary Material online). Critically, however, catalytically important surface exposed regions, such as the binding sites for cytochrome *c* on OXPHOS complexes III and IV, have remained devoid of MS-OPs (supplementary fig. S1*f* and *i*, Supplementary Material online). Nevertheless, in their peripheral position MS-OPs may be involved in the oligomerization of individual OXPHOS complexes into either hetero-oligomeric respiratory supercomplexes (Boekema and Braun 2007) or homo-oligomers. The dimerization of complex V (ATP synthase) via subunits e and g is an illustrative example of the latter (Wagner et al. 2009). As outlined above, however, even if an MS-OP is currently involved in dimerization, this does not necessarily imply that it initially evolved for that purpose. And as for MS-RPs, the evolutionary origins of MS-OPs have thus far remained obscure.

Finally, it is important to note that no substantial constructive evolution can be discerned in the bioenergetic complexes of chloroplasts: As is the case for the chloroplast ribosome (Sharma et al. 2007), the composition and structure of chloroplast photosystems I and II, ATP synthase, and the cytochrome *b_6_f* complex is very similar to that of bacteria (Berry 2003). This contrasts sharply with the genome content of mitochondria and chloroplasts, which has evolved convergently by retaining a similar set of genes for ribosomal proteins and bioenergetic proteins (Maier et al. 2013).

### Cryo-EM Structure of an rRNA-Rich Mitochondrial Ribosome

In order to obtain structural insights into the constructive evolution of mitoribosomes, we determined the cryo-EM structure of a mitoribosome with a nonreduced amount of rRNA: The 73S mitoribosome from the filamentous fungus *N. crassa* (Michel et al. 1977; Neupert et al. 1979). The 73S mitoribosome consists of 77 M-RPs (47 B-RPs and 30 MS-RPs) and two large rRNAs (it lacks 5S rRNA) (supplementary table S5, Supplementary Material online). And assuming that its MTSs are similar in length as those of *Saccharomyces cerevisiae*, its estimated total molecular mass is approximately 3.7 MDa. Here, we will focus on those features of the 73S that are relevant for its structural evolution.

**Fig. 2.—:**
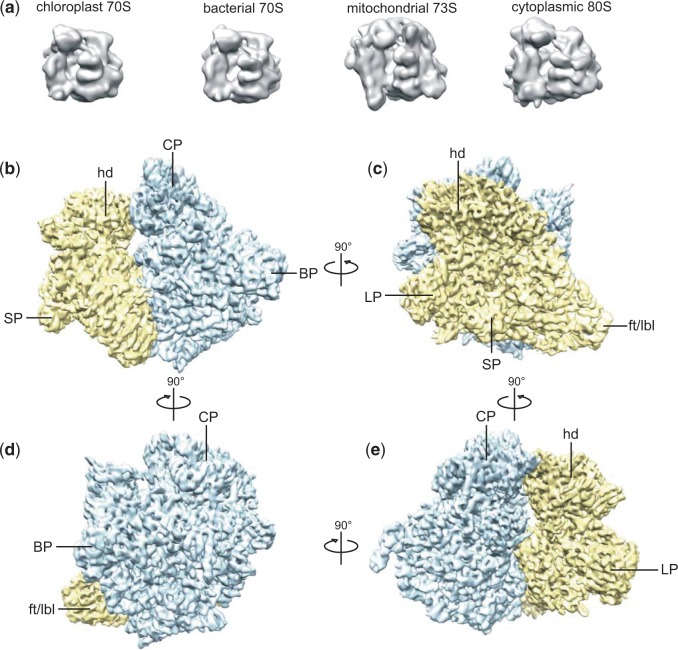
Overall structure of the *N. crassa* 73S mitoribosome*.* (*a*) Gallery of low resolution structures of ribosomes from *Sp. oleracea* chloroplasts (EMDB1417), *T. thermophilus* (EMDB2277), *N. crassa* mitochondria, and the *S. cerevisiae* cytoplasm (EMDB2275), respectively. A comparison with the mammalian 55S mitoribosome was not possible because the electron density map is not publicly available. (*b–e*) Side views the 73S *N. crassa* mitoribosome, rotated in four steps of 90° around the vertical axis. The SSU is depicted in yellow, the LSU in blue. Conserved ribosomal landmarks on the SSU (hd, head; ft/lbl, foot/lower body lobe) and the LSU (CP, central protuberance), as well as unique 73S features (SP, small protuberance; LP, large protuberance; BP, back protuberance) are indicated.

The diameter of the 73S (>300 Å) is significantly larger than that of the bacterial 70 S ribosome (fig. 2*a*), similar to that reported for the mammalian 55S mitoribosome and the *S. cerevisiae* 72S (Sharma et al. 2003; Amunts et al. 2014). But unlike the porous 55S however, the core of the 73S is as compact as that of the 70S. The overall structure of the 70S and the 73S is very alike, and landmark ribosomal features such as the head (hd) and foot (ft) of the SSU, a widened central protuberance (CP) (fig. 2*b*–*e*) and the polypeptide exit tunnel of the LSU (fig. 4*e* and *f*) are clearly distinguishable. Also the E-site of the 73S appears to be completely intact (data not shown), in contrast to what has been reported for the 55S (Sharma et al. 2003; Nierhaus 2006).

**Fig. 3.—:**
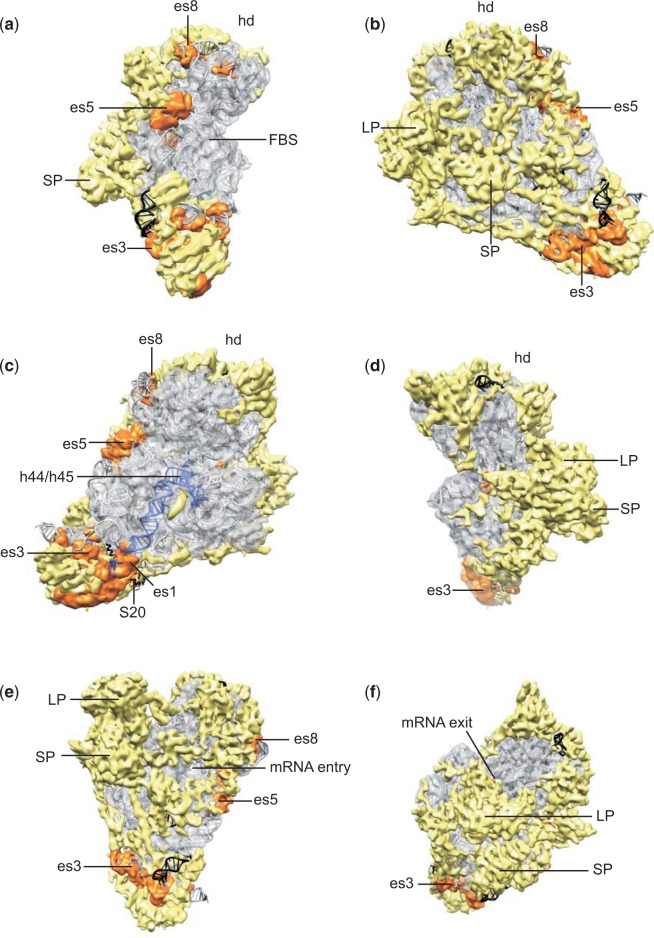
Constructive evolution of the mitoribosomal SSU. Electron density orthologous to *E. coli* rRNA segments and ribosomal proteins is represented in transparent gray, rRNA expansion segments in orange, and the MS-RP density in yellow. The *E. coli* crystal structure is depicted in gray ribbons underneath, with rRNA segments and proteins lacking from the 73S depicted in black. Ribosomal RNA helices h44 and h45 in (*c*) are depicted in magenta. The views of panel (*a–d*) correspond to those of figure 2*b***–***e*; panels (*e* and *f*) are views from the solvent side into the mRNA entry- and exit channel, respectively. Ribosomal landmarks are indicated as in figure 2, FBS, factor binding site; expansion segments are indicated with “es.”

**Fig. 4.—:**
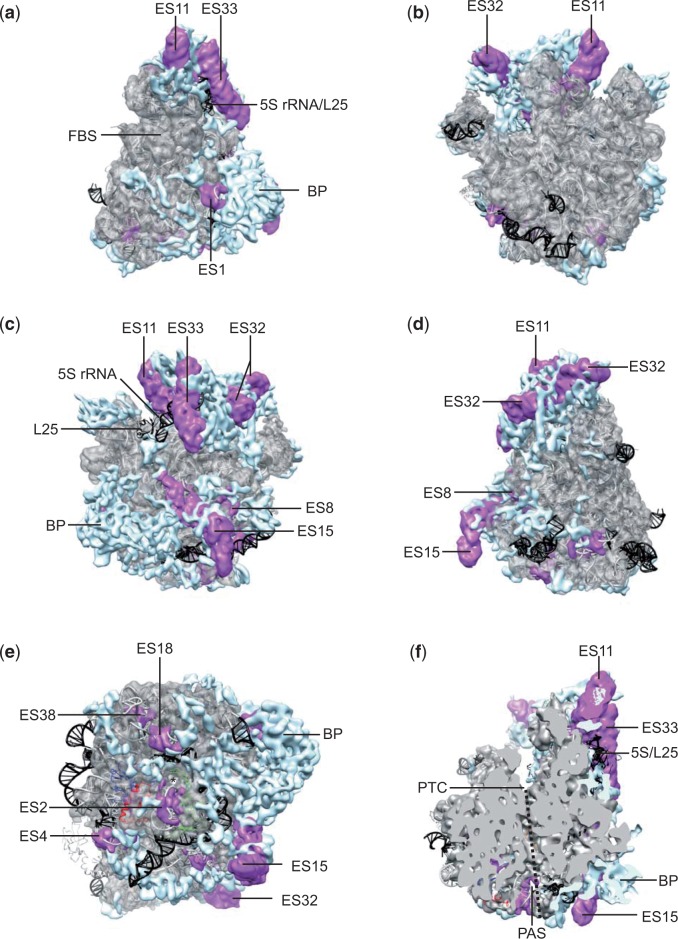
Constructive evolution of the mitoribosomal LSU. Electron density orthologous to *E. coli* rRNA segments and ribosomal proteins is represented in transparent gray, rRNA expansion segments in magenta, and MS-RP density in blue. The *E. coli* crystal structure is depicted in gray ribbons underneath, with rRNA segments and proteins lacking in the 73S in black. The views of panels (*a***–***d*) correspond to those of figure 2 *b***–***e*; panel (*e*) is a view into the polypeptide exit tunnel (*) from the membrane region, with *E. coli* ribosomal exit site proteins L23, L24, and L29 colored in blue, green, and red, respectively. Panel (*f*) is a cut-away view similar to (*a*), illustrating the polypeptide exit tunnel with a dotted line. The PAS viewed from inside the polypeptide exit tunnel is indicated. Ribosomal landmarks are indicated as in figure 2; FBS, factor binding site; PTC, peptidyl transferase center; expansion segments are indicated with “ES.”

A small number of unique features can be distinguished in the overall structure of the 73S. The most prominent are two unevenly sized protuberances at the back of the central domain of the SSU (large and small protuberance: “SP” and “LP,” figs. 2*b*, *c*, *e*, and 3), and one on the lower back region of the LSU (back protuberance: “BP,” figs. 2*d* and 4). The location of the LP close to the mRNA exit channel (fig. 3*f*), may indicate a role in mRNA processing. Further, the BP together with the extended foot region of the SSU, which resembles the “lower body lobe” of the 55S (Sharma et al. 2003), may be involved in membrane binding. An enlarged membrane interacting region is not unique for mitoribosomes, though, as it has previously been observed in cytoplasmic ribosomes from rat liver (Dube et al. 1998). Moreover, as we will outline in more detail below, such presumed “location based” functions of the mitochondrion-specific protuberances may only be secondary functions: Their primary function may be different.

One of the remarkable features of mitoribosomes (with the exception of plants, see supplementary table S1, Supplementary Material online) is the absence of 5S rRNA. In the CP of the 73S mitoribosome, two of the three proteins that connect the 5S to the 23S rRNA (uL18 and uL25) are also absent. The 73S structure reveals that the upper half of the 5S rRNA is physically replaced by rRNA expansion segment ES33 (fig. 4*a* and *c*). The neighboring ES11 and ES32 are responsible for the widening of the 73S CP, relative to that of the 70S. Interestingly, the mechanism by which the mammalian 55S mitoribosome deals with the loss of 5S rRNA is fundamentally different: Although approximately the same region of the 5S is physically replaced, the replacement is mostly mediated by r-protein rather than rRNA (Sharma et al. 2003). The 55S electron density previously designated as “LSU handle” (MRPL52 according to the higher resolution 55S structure [Greber et al. 2014]) adopts roughly the same position as ES33 of the 73S. Taken together, these findings indicate a high degree of structural plasticity of the ribosome, by which it can cope with the loss of a major structural component.

We also observe the “polypeptide accessible site” (PAS: fig. 4*f*), which has been proposed to form a mitochondrion-specific alternative exit site for nascent membrane proteins (Sharma et al. 2003). However, a similar albeit slightly narrower site can be observed in the bacterial LSU (Seidelt et al. 2009), indicating that it is not unique for mitoribosomes. In view of the approximately 90° bend that a polypeptide would have to adopt in order to make use of the PAS, we consider it highly unlikely that this pathway is used by any nascent chain.

### Constructive Evolution of the Mitoribosome

For visualizing the constructive evolution of the mitoribosome, we docked atomic models of the bacterial ribosome into our experimental electron density, subtracted orthologous protein and RNA density, and separated the remaining electron density into rRNA (i.e., expansion segments) and protein (i.e., MS-RPs and extensions of B-RPs) (figs. 3 and 4). This analysis reveals that the core of the 73S is virtually identical to the bacterial ribosome, and that the newly recruited protein mass is distributed as an interconnected peripheral network over nearly the entire ribosomal surface. One prominently recurring feature within the mitochondrion-specific protein density are long alpha-helical regions on the ribosomal surface, in particular on the back of the SSU (fig. 3*b*). At least one of these long helical regions is also visible in the mammalian 55S mitoribosome (Sharma et al. 2003), and might thus represent one of the 15 ubiquitously distributed MS-RPs (Smits et al. 2007). At this resolution though, and in the absence of high resolution structures of MS-RPs from the SSU, it is not possible to unambiguously identify individual MS-RPs. Notably, the entire catalytic core of the ribosome, that is, the subunit interface of both subunits, the peptidyl transferase- and decoding centers (figs. 3*c* and 4*b*), as well as the initiation/elongation factor binding site (figs. 3*a* and 4*a*), are completely devoid of MS-RPs. The only exception is a small proteinaceous electron density sandwiched between h44 and h45 of the SSU just below the decoding center (fig. 3*c*).

In summary, on a structural level the constructive evolution of mitochondrial ribosomes resembles that of OXPHOS complexes (I, III, IV, and V) in the following three aspects: 1) structural conservation of the core region; 2) peripheral distribution of mitochondrion-specific proteins; and 3) exclusion of mitochondrion-specific proteins from catalytically important surfaces. Thus, both compositionally and structurally, mitochondrial ribosomes and OXPHOS complexes have evolved very similarly via a constructive phase early in eukaryote evolution and a reductive phase during metazoan evolution.

### Did Deleterious Mitochondrial Mutations Drive the Recruitment of Novel Proteins?

As outlined above, the reductive phase in the evolution of mitochondrial ribosomes and OXPHOS complexes can be explained by a particular property of metazoan mitochondrial genomes: A deletion bias. Here, we will argue that their constructive phase can be explained by a more general property of mitochondrial genomes, namely the relatively high rate at which they accumulate (slightly) deleterious mutations (Neiman and Taylor 2009). Because this property is conserved across the eukaryotic domain (Lynch and Blanchard 1998), it was most likely present in the last eukaryotic common ancestor where most of the recruitment of MS-RPs and MS-OPs occurred.

First, all the mitochondrial complexes that have commonly retained at least one mtDNA encoded structural gene, that is, the mitoribosome and OXPHOS complexes I, III, IV, and V, have all undergone constructive evolution (supplementary table S6, Supplementary Material online). On the other hand, highly conserved mitochondrial enzymes that lack mitochondrial genes such as those of the tricarboxylic acid (TCA) cycle, did not undergo constructive evolution (Huynen et al. 1999). OXPHOS complex II is an even more striking example of a strict relationship between mitochondrial gene products and constructive evolution: It lacks MS-OPs in fungi and metazoa where complex II is exclusively nuclear encoded, but it does contain (lineage-specific) MS-OPs in plants (Millar et al. 2004), where mitochondrial complex II genes are also common (Adams et al. 2001). In other words, during fungal/metazoan evolution the complex II genes “escaped” to the nucleus before any MS-OPs were recruited, whereas during plant evolution their retention by the mitochondrial genome lead to MS-OPs recruitment, similarly to the other four OXPHOS complexes. Note however, that genes may still be transferred from the mitochondrion to the nucleus after a given complex has recruited an MS-OP. Examples of this can also be found in complex II of plants, where gene transfer is known to be an ongoing process (Adams et al. 2001): Although in *Solanum tuberosum* the mitochondrial copy of *sdh4* remains functional (Siqueira et al. 2002), in *Pisum sativum* and *Arabidopsis thaliana* the mitochondrial *sdh4* copy has eroded into a pseudogene (Giege et al. 1998; Kubo and Kadowaki 2001), and a functional copy is now encoded in the nuclear genome (Kubo and Kadowaki 2001; Figueroa et al. 2002). Such a scenario can also explain the presence of several MS-OPs in complex II of Trypanosoma (Morales et al. 2009), even though all subunits are currently encoded in the nucleus.

Second, it is well-described how (slightly) deleterious mutations can catalyze the recruitment of novel proteins, through either of two related evolutionary mechanisms: “constructive neutral evolution (CNE)” (Stoltzfus 1999; Lukes et al. 2011) or “random genetic drift followed by secondary selection” (Fernandez and Lynch 2011). Although the order of events differs among the two mechanisms, that is, mutation preceding the recruitment or vice versa, the end result of both mechanisms is the same: A deleterious mutation(s) in gene *a* renders protein/RNA A structurally (and thus functionally) dependent on novel interactor B. This dependence can subsequently increase in a ratchet-like fashion upon continued mutation accumulation, and in the resulting A:B complex, B effectively compensates for the mutational effect(s) on A. Mitoribosomes and OXPHOS complexes have both been suggested as potential products of such a mechanism (Gray et al. 2010; Lukes et al. 2011; Huynen et al. 2013); however, protein recruitment to the former was thus far consistently discussed in relation to rRNA reduction. We emphasize here that most of the recruitment took place long before the reduction of rRNA.

Taken together, we postulate that early in eukaryote evolution (i.e., before divergence of the major eukaryotic groups) mitochondrially encoded rRNAs and OXPHOS genes accumulated a large number of (slightly) deleterious mutations, which was accompanied by the recruitment of an initial set of universally conserved MS-RPs and MS-OPs. Yet, we cannot distinguish between CNE and random genetic drift followed by secondary selection. Subunit recruitment then continued in the different lineages of eukaryotes, explaining why both mitochondrial ribosomes and OXPHOS complexes currently contain a mixture of universally conserved and lineage-specific MS-RPs/MS-OPs. The primary function of these MS-RPs and MS-OPs is to provide structural compensation for the destabilizing mutational effects on the mitochondrially encoded rRNAs and OXPHOS proteins. Although such a compensatory roles most likely provided selective advantages that were subject to adaptive evolution, we emphasize that the recruitment mechanisms crucially involved (slightly) deleterious mutations, and were hence nonadaptive in nature.

### Evidence for (Slightly) Deleterious Mutations in Mitochondrial Gene Products

If the constructive evolution of mitochondrial ribosomes and OXPHOS complexes was indeed driven by (slightly) deleterious mutation accumulation in mitochondrial genes, then it should be possible to detect mutational effects in the sequences and/or products of those genes in contemporary eukaryotes. Crucially, such effects are expected to be absent from analogous complexes that did not undergo constructive evolution, such as the ribosome and the cytochrome *b_6_f* complex of chloroplasts.

The stability of ribosomes is largely determined by rRNA basepairing, followed by protein:rRNA interactions and to a lesser extent by protein:protein interactions. Only the former of these three interactions can be readily estimated from genomic sequences, and hence we analyzed the relative stability of rRNAs from sequences and secondary structure alignments available from the Comparative RNA Website (Cannone et al. 2002). Destabilization of rRNA has previously been used as an indicator for deleterious mutation accumulation in endosymbiotic bacteria (Lambert and Moran 1998). Indeed, a previously published estimate of the total number of base pairs in a handful of model organisms (supplementary fig. S2, Supplementary Material online) indicates that nonmetazoan (i.e., nonreduced) mitochondrial rRNAs contain significantly fewer base pairs than bacterial and chloroplast rRNAs (Fields and Gutell 1996). Although the differences may not seem dramatic, even point mutations in rRNAs can have severe consequences on ribosomal function (Lee et al. 1997).

**Fig. 5.—:**
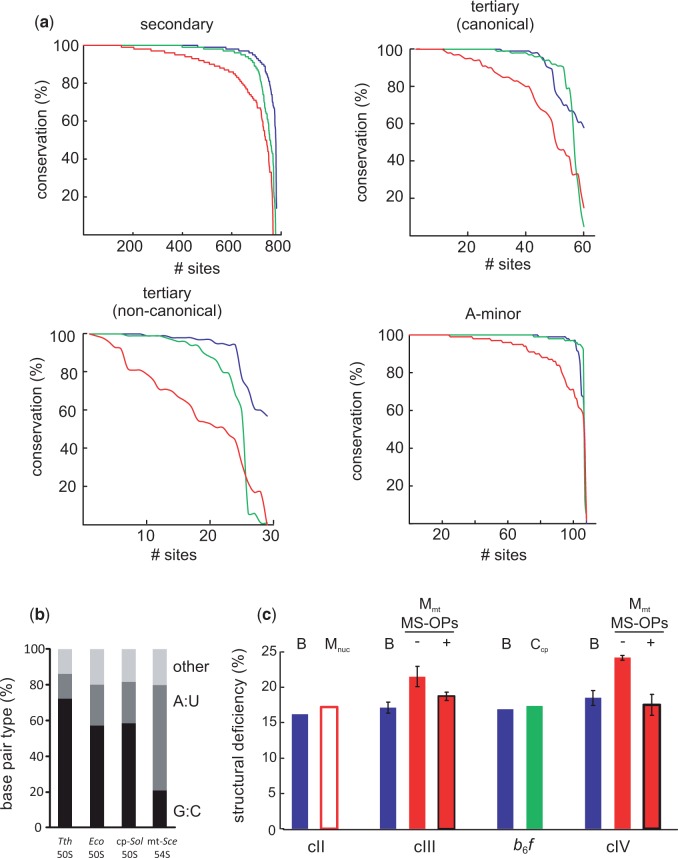
Mitochondrially encoded rRNAs and OXPHOS proteins are relatively unstable. (*a*) The relative stability of mitochondrial rRNAs was estimated from the levels of base pairing. Red, mitochondria; blue, bacteria; green, chloroplasts. Base pairing conservation values (in %) for secondary, canonical tertiary, and noncanonical tertiary, and A-minor interaction sites were derived from an alignment of hundreds of rRNAs. (*b*) Analysis of base pairing types within the conserved rRNA core of the LSU. G:C base pairs are indicated in black, A:U base pairs in dark gray, and other base pairs in light gray. (*c*) The structural deficiency of OXPHOS proteins was expressed as the fraction of underprotected backbone hydrogen bonds (in %, ± standard deviation). Values were calculated from available crystal structures of the (combined) mitochondrially encoded subunits and their bacterial/chloroplast counterparts, either in absence (−) or in presence (+) of MS-OPs. B, bacterial; M_nuc_, nuclear-encoded mitochondrial subunits; M_mt_, mitochondrially encoded mitochondrial subunits; C_cp_, chloroplast-encoded subunits; cII, complex II; cIII, complex III; *b*_6_*f*, cytochrome *b*_6_*f* complex; cIV, complex IV.

For a more extended analysis, we separately analyzed the conservation levels of four different types of base pairs, that is, secondary, canonical tertiary, noncanonical tertiary, and secondary A-minor interaction sites (fig. 5*a*), based on alignments of hundreds of rRNA sequences. (Note that this analysis is not influenced by the length reduction of rRNA in metazoa, as conservation levels are only calculated when both nucleotides of an interaction site are present). For all four types of base pairs, the conservation levels in mitochondria are much lower than those in chloroplasts, which in turn are very similar to those of bacteria. This shows that reduced base pairing is a unique property of mitochondrial rRNAs that applies to all four different types of interaction sites. Hence, mitochondrial rRNAs in general contain fewer base pairs, and are thus inherently less stable than their bacterial and chloroplast counterparts. As argued elsewhere on population genetic grounds (Lynch 1996), and given the high degree of structural conservation of the mitoribosomal core shown here, it is unlikely that this destabilization can be overcome by novel (lineage-specific) compensatory base pairs.

Next, we reasoned that not only the number of base pairs, but also the type of base pairing may influence mitoribosomal RNA stability. Based on the inverse correlation between the A/U content of rRNA and the optimum growth temperature of bacteria, it is thought that a higher level of G:C base pairing is required for rRNA (thermo-)stability. Because mitochondrial genomes and rRNAs generally have a very low G:C content (Lang et al. 1999), we analyzed the levels of G:C and A:U base pairing within the 83 RNA helices that are conserved among the large ribosomal subunits of *T. thermophilus*, *E. coli*, the *S. cerevisiae* mitochondrion, and the *Sp. oleracea* chloroplast. Even though there is no high resolution structure available from the latter, cryo-EM studies have shown that its overall structure is very similar to that of *E. coli* (Manuell et al. 2007; Sharma et al. 2007). Indeed, the thermophilic bacterium *T. thermophilus* has a much higher fraction of G:C base pairs than *E. coli* (72% vs. 57%), in agreement with its higher growth optimum (fig. 5*b*). The chloroplast LSU differs only marginally from *E. coli* with 58% G:C base pairs. The 54S mitoribosomal LSU on the other hand contains merely 21% G:C base pairs, despite growing at a only a slightly lower temperature than *E. coli.* This decrease in G:C base pairs is accompanied by a proportional increase in A:U base pairs, and results in a loss of approximately 260 hydrogen bonds relative to *E. coli.* Thus, mitoribosomal RNA is not only destabilized by a decrease in the number of base pairs but also by a larger proportion of weaker base pairs, compared to bacteria. A similar effect, albeit much less dramatic, has been observed in endosymbiotic bacteria and is most likely a direct consequence of the overall high A/T content of mitochondrial/endosymbiotic genomes (Lambert and Moran 1998). Importantly, the evolutionary mechanisms underlying the A/T mutation bias are generally also considered being nonadaptive in nature (Lynch 2007b).

Unlike ribosome stability, the relative stability of proteins cannot be readily approximated from genomic sequences; however, it can be estimated from experimentally determined atomic models. Hence, for OXPHOS complexes we estimated their relative stability from available crystal structures by calculating their “structural deficiency,” that is, the fraction of underprotected backbone hydrogen bonds (Fernandez and Lynch 2011; see Materials and Methods). In stably folded proteins most backbone hydrogen bonds are well-protected by neighboring nonpolar groups; an increased number of underprotected backbone hydrogen bonds is an indicator of reduced protein stability (Fernandez and Scott 2003). It has previously been shown that the structural deficiency of proteins from endosymbiotic bacteria is generally higher than that of free-living bacteria (Fernandez and Lynch 2011), in agreement with the above mentioned destabilization of rRNA in endosymbionts (Lambert and Moran 1998). First, we calculated the structural deficiency of the mitochondrially encoded subunits of complexes III and IV in the absence of MS-OPs. Indeed, their structural deficiency is significantly higher than that of their bacterial counterparts (fig. 5*c*, red bars without outline vs. blue bars), which is indicative of a lower overall stability. On the other hand, no signs of destabilization could be observed in the nuclear-encoded subunits of complex II (fig. 5*c*, white bar “M_nuc_” vs. blue bar). Unfortunately, high resolution structural information is lacking for the mitochondrially encoded subunits of complexes I and V, as well as for plant complex II. But importantly, as observed with the rRNAs, destabilization is unique for mitochondrially encoded components, as the structural deficiency of the chloroplast-encoded subunits of the cytochrome *b_6_f* complex is similar to that of their bacterial counterparts (fig. 5*c* “*b*_6_*f*,” green bar vs. blue bar).

Next, we investigated the above-hypothesized compensatory nature of MS-OPs by including them in the calculation of structural deficiency. For the mitochondrially encoded subunits of both OXPHOS complexes III and IV, the presence of MS-OPs indeed reduces their structural deficiency, which now approaches the levels observed for their bacterial counterparts (fig. 5*c*, red bars with black outline vs. blue bars). This indicates that at least the MS-OPs of OXPHOS complexes III and IV have a stabilizing or compensatory effect on the relatively unstable mitochondrially encoded subunits of those complexes.

Taken together, these data show that the mitochondrially encoded components of mitochondrial ribosomes and OXPHOS complexes are inherently less stable than their bacterial and chloroplast counterparts. Furthermore, for OXPHOS complexes III and IV we could indeed show that MS-OPs have a compensatory effect. The compositional and structural similarities in the evolution of complexes I and V as well as the mitoribosome strongly suggest similar compensatory functions for MS-OPs and MS-RPs in the latter. These findings, in combination with the arguments and literature cited above, strongly support our hypothesis that (slightly) deleterious mitochondrial mutations indeed drove the recruitment of novel proteins to mitochondrial ribosomes and OXPHOS complexes.

## Discussion

When the biophysical properties of mitoribosomes were first described several decades ago, the results were received with skepticism because of the large and unexpected deviations from bacterial ribosomes. Meanwhile, the deviant structure of mitoribosomes has become generally accepted, yet, the evolutionary causes and functional consequences of it have thus far remained unclear. Although the prosthetic hypothesis (see Introduction) has gained great awareness, this intuitive structural concept has been refuted on the basis of a cryo-EM structure (Sharma et al. 2003). It was then proposed that the elevated protein content of mitoribosomes would play a functional, rather than a structural role. While at first sight this proposal may seem plausible, it does not address the evolutionary origin of the high protein content and may therefore represent an incomplete view.

**Fig. 6.—:**
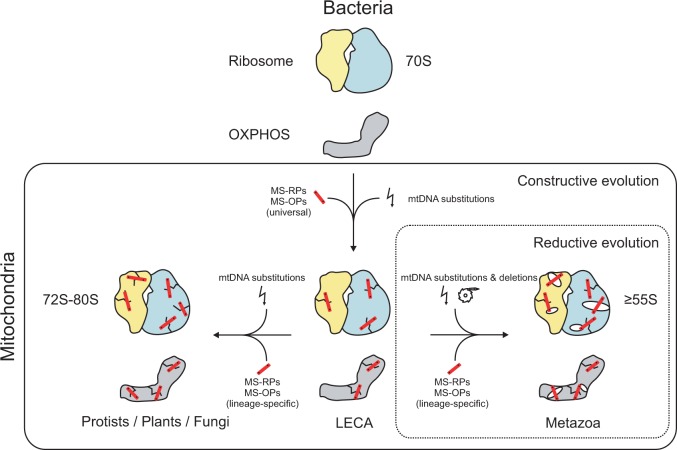
Simplified model for the parallel structural evolution of mitochondrial ribosomes and OXPHOS complexes. Early in eukaryote evolution, mitochondrial ribosomes (yellow/blue) and OXPHOS complexes (gray) underwent a phase of constructive evolution, during which numerous nuclear encoded mitochondrion-specific proteins (MS-RPs and MS-OPs: red), were recruited. The function of these newly recruited proteins is to provide structural compensation to the destabilizing effect of deleterious mutations (lightning symbol, yielding cracks) that accumulated in mtDNA-encoded rRNAs and OXPHOS proteins. A subsequent phase of reductive evolution, during which mitochondrially encoded rRNAs and OXPHOS proteins were gradually reduced in length (ratchet symbol), was unique for metazoa and led to the reduced sedimentation coefficient and high porosity (holes) of the mammalian 55S mitoribosome, and the loss of transmembrane domains from complex I. Mitoribosomes and OXPHOS complexes of plants, fungi and protists on the other hand, have remained more similar to those of the inferred last eukaryotic common ancestor (LECA).

Here, we have analyzed the deviant structure of mitoribosomes in an evolutionary context, which has yielded profound novel insights with broad explanatory power. We have revealed that the structural evolution of mitoribosomes was not a unique process but a rather general one that has clear parallels in mitochondrial OXPHOS complexes. In particular, we have shown that the structures of mitoribosomes and OXPHOS complexes have evolved very similarly via two discrete phases (fig. 6): 1) A constructive phase early in eukaryote evolution, during which dozens of novel ribosomal- and OXPHOS proteins (MS-RPs and MS-OPs, respectively) were recruited, and 2) a reductive phase specific to metazoan evolution, during which mitochondrially encoded rRNAs and OXPHOS proteins were gradually reduced in length.

As outlined above, the occurrence of both the constructive and the reductive phase can be explained by (slightly) deleterious mutations and deletions in mitochondrial genomes, respectively. And thus, the origins of the deviant structure of mitochondrial ribosomes and OXPHOS complexes must be sought in the (population) genetic characteristics of mitochondrial genomes, rather than in altered demands for (the regulation of) translation or OXPHOS. The causes of deleterious mutation accumulation in mitochondrial genomes, which we argue underlay the constructive phase, have been established and discussed extensively, and include 1) high mutation rates; 2) small effective population sizes; and 3) low rates of recombination (Neiman and Taylor 2009). In chloroplast genomes on the other hand, deleterious mutations accumulate much slower than in mitochondria (Lynch 1997), explaining why virtually no constructive evolution can be discerned in the photosynthetic apparatus and ribosomes of chloroplasts. Although we cannot formally exclude alternative (e.g., adaptive) recruitment mechanisms for the MS-RPs/OXPHOS proteins, our concept involving deleterious mutations in the mitochondrial genome is supported by 1) well-established recruitment mechanisms; 2) population genetic data, and 3) signs of destabilization in mitochondrial genes. But perhaps most importantly, the single concept outlined here can explain the recruitment of all approximately 75 MS-OPs/MS-RPs.

The mutation rate in plant mitochondrial genomes is known to be exceptionally low, and the amount of MS-RPs in plants also appears to be lower than in other eukaryotes (fig. 1). Thus, at first sight this may seem to provide additional support for the involvement of deleterious mutations in the recruitment of MS-RPs. However, this trend is not observed in OXPHOS complexes: The amount of MS-OPs does not appear to be significantly lower in plants than in other eukaryotes (supplementary table S6, Supplementary Material online). The most likely explanation for this discrepancy is that unlike plant OXPHOS complexes, the plant mitoribosomal proteome has thus far not been characterized experimentally, and hence the amount of MS-RPs depicted in figure 1 is most likely an underestimate due to the absence of plant-specific MS-RPs. For similar reasons, we refrain from directly relating mitochondrial mutation rates to the amount of MS-RPs/MS-OPs in different lineages.

The causes of the deletion bias in metazoan mitochondrial genomes, which we argue underlay the reductive phase, are less well understood, even though deletion bias is known to be a common genomic phenomenon that needs to be countered by selection (Mira et al. 2001). To our knowledge, the only attempt to explain the exceptionally high reductive pressure on metazoan mitochondrial genomes in particular is the so-called “mutation pressure hypothesis” (Lynch et al. 2006), according to which the contraction (or expansion) of genomes is determined by the relative powers of mutation and random genetic drift. But regardless of the exact population genetic determinants, from our comparative genomic analyses it is obvious that extreme genome reduction is unique for metazoa.

Based on the common structural principles and the common recruitment mechanism that we have described for the approximately 75 MS-OPs and MS-RPs, we propose that these proteins share a common primary function: Namely, to provide structural compensation to core components that were “damaged” by deleterious mitochondrial mutations (as shown above for the MS-OPs of OXPHOS complexes III and IV, fig. 5*c*). Metaphorically, this compensatory structural function that we propose is comparable to the “propping up” of a deteriorating building. The damage, and thus the compensation, may have occurred on either of three levels: Biogenesis (e.g., folding and assembly), stability, or activity, explaining why 1) OXPHOS complexes that lack MS-OPs are often disturbed on any of these levels, and 2) why MS-OPs are commonly found to be involved in assembly, stability, and regulation of OXPHOS complexes (Berry 2003; Zickermann et al. 2010; Angerer et al. 2011). The few available functional data on MS-RPs point toward similar functions in mitoribosomes (Kaur and Stuart 2011). The importance of ribosomal proteins for ribosome assembly has been stressed elsewhere, in relation to the retention of a core set of B-RPs in organelle genomes (Maier et al. 2013).

Note however, that MS-OPs and MS-RPs not only provide compensation to mutations that have led to their initial recruitment, but also to mutations that occurred later. The genetic relationship between nuclear-encoded proteins and mitochondrial proteins/rRNAs is asymmetric (Rand et al. 2004; Osada and Akashi 2012; Barreto and Burton 2013) in the sense that compensatory mutations in the former continue to counteract the effects of deleterious mutations in the latter. Hence, we speculate that the high mutation pressure on mitochondrial genomes may only be tolerated by eukaryotes by virtue of their MS-OPs and MS-RPs, which are capable of providing continued structural compensation. Accordingly, one experimentally testable prediction is that mitochondrion-specific proteins with similar compensating structural roles should also be present in other macromolecular complexes with mitochondrially encoded components, such as the SecY complex and RNA polymerase from *Reclinomonas americana* (Lang et al. 1997).

It is worthwhile to address the “raw material” that may have served as a source of novel proteins for OXPHOS complexes and the mitoribosome. Examples have been given for the recruitment of complete enzymes to mitochondrial ribosomes (Smits et al. 2007) and OXPHOS complexes (Gabaldon et al. 2005), most famously the mitochondrial processing peptidase that became an integral component of complex III (Braun and Schmitz 1995). Additionally, we propose that MTSs may have been recruited frequently, because 1) the positively charged amino-terminal region of MTSs are likely to interact fortuitously with the negatively charged rRNA of the mitoribosome; 2) hydrophobic segments that are released after cleavage of bipartite MTSs are likely to interact with OXPHOS complexes in the 2D plane of the inner mitochondrial membrane; 3) the topology of all MS-OPs (Zickermann et al. 2010) (see Footnote) (N_in_-C_out_) is identical to that of the hydrophobic segment released from bipartite MTSs (Herrmann and Hell 2005). One known example of this scenario is subunit 9 from mammalian complex III, which is the (hydrophilic) MTS of the Rieske Iron Sulfur protein that resides in the same complex (Iwata et al. 1998).

In summary, we have provided a general concept that provides simple explanations for the deviant structure of mitochondrial ribosomes and OXPHOS complexes. Although we emphasize here a primary structural role for MS-OPs and MS-RPs, we do not exclude that individual MS-RPs and MS-OPs may indeed have secondary functions in mitochondrion-specific features of translation or OXPHOS, as suggested elsewhere. For mitoribosomes these could include: Membrane binding; the interaction with message-specific translational activators; and tissue-specific expression (Pagliarini et al. 2008). For OXPHOS complexes these could also include tissue-specific expression (Pagliarini et al. 2008); the oligomerization into respiratory supercomplexes (Boekema and Braun 2007); or the dimerization of ATP synthase (Wagner et al. 2009). By inducing membrane curvature (Strauss et al. 2008), the latter may have contributed to the increase in surface area of the mitochondrial inner membrane, and thus to an increase of mitochondrial power (Lane and Martin 2010). Hence, the constructive evolution of mitochondrial ribosomes and OXPHOS complexes may have opened up previously inaccessible but crucial evolutionary paths throughout mitochondrial and thus eukaryotic evolution.

### Footnote

Note that mitochondrial Atp8/A6L, with N_out_-C_in_ topology, is orthologous to bacterial AtpF, and thus neither an MS-OP nor an exception in the group of single transmembrane domain MS-OPs as mentioned in (Zickermann et al. 2010).

## Supplementary Material

Supplementary tables S1–S6 and figures S1 and S2 are available at *Genome Biology and Evolution* online (http://www.gbe.oxfordjournals.org/).

Supplementary Data
