# Supplementary material for: Parallel Structural Evolution of Mitochondrial Ribosomes and OXPHOS Complexes
Source: Genome Biol Evol. 2015 Apr 9;7(5):1235–51. doi: 10.1093/gbe/evv061 (PMC4453056; doi:10.1093/gbe/evv061)
Supplement: Supplementary Data [file supp_7_5_1235__index.html]

Parallel structural evolution of mitochondrial ribosomes and OXPHOS complexes — Parallel Structural Evolution of Mitochondrial Ribosomes and OXPHOS Complexes — Supplementary Data 

# Parallel Structural Evolution of Mitochondrial Ribosomes and OXPHOS Complexes

## Supplementary Data

files

**Files in this Data Supplement:**

- Supplementary Data - pdf file
- Supplementary Data - pdf file
- Supplementary Data - pdf file
- Supplementary Data - pdf file
