# Supplementary material for: Parallel Structural Evolution of Mitochondrial Ribosomes and OXPHOS Complexes
Source: Genome Biol Evol. 2015 Apr 9;7(5):1235–51. doi: 10.1093/gbe/evv061 (PMC4453056; doi:10.1093/gbe/evv061)
Supplement: Supplementary Data [file supp_evv061_Vandersluis_supplementary.pdf]

Supplementary Information

Supplementary Results

Comparative genomics of MS-OPs from OXPHOS complexes III and IV

We gathered published biochemical evidence for the number of unique subunits in complex III and IV (Supplementary Tables II and III), and analyzed their presence in nine eukaryotic model organisms (Supplementary Table IV) with aid of the COG (Tatusov et al. 2003), eggNOG (Jensen et al. 2008), STRING (Snel et al. 2000) and PhylomeDB (Huerta-Cepas et al. 2008) databases, as well as iterative BLAST searches. From these analyses it can be inferred that the mitochondrion of the last eukaryotic common ancestor (LECA) contained at least two MS-OPs in complex III (Core 1 and Core 2) and one MS-OP in complex IV (Subunit Vb). However, given the minimal number of seven unique proteins in all the biochemically characterized complexes III and IV (Supplementary Tables II & III), it seems more likely that complexes III and IV both contained at least four MS-OPs in the LECA. Note that subunit CoxIV of bacterial complex IV lacks a counterpart in mitochondria. The discrepancy between both analyses can be explained by the small size and the notoriously low levels of sequence similarity among integral membrane MS-OPs in particular, which may prevent their identification by sequence similarity searches.

Legend to supplementary Figure 1

Constructive evolution of mitochondrial OXPHOS complexes

Crystal structures of fungal complex I (**a**), chicken complex II (**b**), and bovine complexes III (**d-f**), IV (**g-i**) and V (**c**) were separated into "core" subunits of bacterial descent (grey) and mitochondrion-specific OXPHOS proteins (MS-OPs: orange) and filtered to ~7 Å resolution. The view in panel **a** is a slice through the membrane region viewed from the matrix side, views **b-e**, **g** & **h** are side views with the matrix facing up, and views **f** and **i** are from the inter-membrane space with bound cytochrome *c* depicted in red. The lack of MS-OPs from the cytochrome *c* binding sites on the (homodimeric) complexes III and IV can be observed in the symmetry related copies. Note that from complex V only fragments from three of the eight MS-OPs have been crystallized. Approximate membrane boundaries are indicated with dashed lines and complexes are not drawn to scale.

Legend to supplementary Figure 2

Decreased base pairing in mitochondrial rRNAs

The predicted (Fields, Gutell 1996) total number of base pairs (± standard deviation) from individual rRNAs of bacteria (B), chloroplasts (C) and non-metazoan mitochondria (M).

Supplementary Table II

Number of experimentally characterized unique subunits in OXPHOS complex III

Organism	# subunits	Reference
Euglena gracilis	10	(Mukai et al. 1989)
Seculamonas ecuadoriensis	7	(Marx et al. 2003)
Crithidia fasciculata	9	(Priest, Hajduk 1992)
Neurospora crassa	8	(Leonard et al. 1981)
Saccharomyces cerevisiae	10	(Hunte et al. 2000)
Arabidopsis thaliana	10	(Eubel, Jansch, Braun 2003)
Arum maculatum	9	(Sunderhaus et al. 2010)
Solanum tuberosum	10	(Braun, Schmitz 1992)
Phaseolus vulgaris	8	(Eubel, Jansch, Braun 2003)
Polytomella spp.	10	(Gutierrez-Cirlos et al. 1994)
Chlamydomonas reinhardtii	9	(van Lis et al. 2003)
Triticum aestivum	10	(Braun et al. 1995)
Bos taurus	11	(Iwata et al. 1998)

Supplementary Table III

Number of experimentally characterized unique subunits in OXPHOS complex IV

Organism	# subunits	Reference
Euglena gracilis	15	(Bronstrup, Hachtel 1989)
Crithidia fasciculata	10	(Speijer et al. 1996)
Neurospora crassa	7	(Weiss, Kolb 1979)
Saccharomyces cerevisiae	11	(Geier et al. 1995)
Arabidopsis thaliana	10	(Millar et al. 2004)
Arum maculatum	9	(Sunderhaus et al. 2010)
Solanum tuberosum	9	(Jansch et al. 1996)
Phaseolus vulgaris	10	(Eubel, Jansch, Braun 2003)
Polytomella spp.	8	(van Lis, Gonzalez-Halphen, Atteia 2005)
Chlamydomonas reinhardtii	10	(van Lis et al. 2003)
Zea mays	8	(Hawkesford, Liddell, Leaver 1989)
Triticum aestivum	7	(Peiffer, Ingle, Ferguson-Miller 1990)
Bos taurus	13	(Tsukihara et al. 1996)

Supplementary Table V

Composition of the Neurospora crassa *73S mitoribosome and the* Escherichia coli *70S ribosome.*

	***E. coli* ribosome**			***N. crassa* mitoribosome**		
	**SSU**	**LSU**	**SSU + LSU**	**SSU**	**LSU**	**SSU + LSU**
Sedimentation	30S	50S	70S	37S	50S	73S
Nucleotides	1542	2904 + 120	4566	1971	3465	5436
B-RPs	21	33	54	19<br>(-S1,-S20)	28<br>(-L18,-L20,-L25,-L33,-L35)	47
MS-RPs	-	-	-	16	14	30
M.W. rRNA (MDa)	0.48	0.93	1.41	0.60	1.06	1.66
M.W. protein (MDa)[a]	0.35	0.44	0.79	1.09	1.18	2.27
M.W. total (MDa)[a]	0.83	1.37	2.20	1.69	2.24	3.93

[a]: including mitochondrial targeting sequences

Supplementary Table VI

Composition of OXPHOS complexes I-V (cI-cV) in bacteria, fungi, mammals and plants.

	Bacteria [a]		***Fungi*** [c]			***Mammals*** [e]			***Plants*** [f]	
		mtDNA	MS-OPs	total	mtDNA	MS-OPs	total	mtDNA	MS-OPs	total
**cI**	14	7[d]	25[d]	**39**[d]	7	31	**45**	9	28	**42**
**cII**	4	-	-	**4**	-	-	**4**	0-1[g]	4	**8**
**cIII**	3[b]	1	7	**10**	1	8	**11**	1	7	**10**
**cIV**	4[b]	3	8	**11**	3	10	**13**	3	7	**10**
**cV**	8	3	11	**19**	2	8	**16**	5	6	**14**
**total**	**33**	**14**	**51**	**83**	**13**	**57**	**89**	**19**	**52**	**84**

[a]: *Escherichia coli* unless stated differently

[b]: *Rhodobacter sphaeroides*

[c]: *Saccharomyces cerevisiae* unless stated differently

[d]: *Yarrowia lipolytica; S. cerevisiae* lacks complex I

[e]: *Bos taurus*

[f]: *Arabidopsis thaliana* unless stated differently

[g]: *Solanum tuberosum*; *A. thaliana* contains a mitochondrial pseudogene of *sdh4 (Giege, Knoop, Brennicke 1998)*.

Supplementary references

Braun, HP, M Emmermann, V Kruft, M Bodicker, UK Schmitz. 1995. The general mitochondrial processing peptidase from wheat is integrated into the cytochrome bc1-complex of the respiratory chain. Planta 195:396-402.

Braun, HP, UK Schmitz. 1992. Affinity purification of cytochrome c reductase from potato mitochondria. Eur J Biochem 208:761-767.

Bronstrup, U, W Hachtel. 1989. Cytochrome c oxidase of Euglena gracilis: purification, characterization, and identification of mitochondrially synthesized subunits. J Bioenerg Biomembr 21:359-373.

Eubel, H, L Jansch, HP Braun. 2003. New insights into the respiratory chain of plant mitochondria. Supercomplexes and a unique composition of complex II. Plant Physiol 133:274-286.

Fields, DS, RR Gutell. 1996. An analysis of large rRNA sequences folded by a thermodynamic method. Fold Des 1:419-430.

Geier, BM, H Schagger, C Ortwein, TA Link, WR Hagen, U Brandt, G Von Jagow. 1995. Kinetic properties and ligand binding of the eleven-subunit cytochrome-c oxidase from Saccharomyces cerevisiae isolated with a novel large-scale purification method. Eur J Biochem 227:296-302.

Giege, P, V Knoop, A Brennicke. 1998. Complex II subunit 4 (sdh4) homologous sequences in plant mitochondrial genomes. Curr Genet 34:313-317.

Gutierrez-Cirlos, EB, A Antaramian, M Vazquez-Acevedo, R Coria, D Gonzalez-Halphen. 1994. A highly active ubiquinol-cytochrome c reductase (bc1 complex) from the colorless alga Polytomella spp., a close relative of Chlamydomonas. Characterization of the heme binding site of cytochrome c1. J Biol Chem 269:9147-9154.

Hawkesford, MJ, AD Liddell, CJ Leaver. 1989. Subunit Composition of Cytochrome c Oxidase in Mitochondria of Zea mays. Plant Physiol 91:1535-1542.

Hunte, C, J Koepke, C Lange, T Rossmanith, H Michel. 2000. Structure at 2.3 A resolution of the cytochrome bc(1) complex from the yeast Saccharomyces cerevisiae co-crystallized with an antibody Fv fragment. Structure 8:669-684.

Iwata, S, JW Lee, K Okada, JK Lee, M Iwata, B Rasmussen, TA Link, S Ramaswamy, BK Jap. 1998. Complete structure of the 11-subunit bovine mitochondrial cytochrome bc1 complex. Science 281:64-71.

Jansch, L, V Kruft, UK Schmitz, HP Braun. 1996. New insights into the composition, molecular mass and stoichiometry of the protein complexes of plant mitochondria. Plant J 9:357-368.

Leonard, K, P Wingfield, T Arad, H Weiss. 1981. Three-dimensional structure of ubiquinol:cytochrome c reductase from Neurospora mitochondria determined by electron microscopy of membrane crystals. J Mol Biol 149:259-274.

Marx, S, M Baumgartner, S Kannan, HP Braun, BF Lang, G Burger. 2003. Structure of the bc1 complex from Seculamonas ecuadoriensis, a jakobid flagellate with an ancestral mitochondrial genome. Mol Biol Evol 20:145-153.

Millar, AH, H Eubel, L Jansch, V Kruft, JL Heazlewood, HP Braun. 2004. Mitochondrial cytochrome c oxidase and succinate dehydrogenase complexes contain plant specific subunits. Plant Mol Biol 56:77-90.

Mukai, K, M Yoshida, H Toyosaki, Y Yao, S Wakabayashi, H Matsubara. 1989. An atypical heme-binding structure of cytochrome c1 of Euglena gracilis mitochondrial complex III. Eur J Biochem 178:649-656.

Peiffer, WE, RT Ingle, S Ferguson-Miller. 1990. Structurally unique plant cytochrome c oxidase isolated from wheat germ, a rich source of plant mitochondrial enzymes. Biochemistry 29:8696-8701.

Priest, JW, SL Hajduk. 1992. Cytochrome c reductase purified from Crithidia fasciculata contains an atypical cytochrome c1. J Biol Chem 267:20188-20195.

Speijer, D, AO Muijsers, H Dekker, A de Haan, CK Breek, SP Albracht, R Benne. 1996. Purification and characterization of cytochrome c oxidase from the insect trypanosomatid Crithidia fasciculata. Mol Biochem Parasitol 79:47-59.

Sunderhaus, S, J Klodmann, C Lenz, HP Braun. 2010. Supramolecular structure of the OXPHOS system in highly thermogenic tissue of Arum maculatum. Plant Physiol Biochem 48:265-272.

Tsukihara, T, H Aoyama, E Yamashita, T Tomizaki, H Yamaguchi, K Shinzawa-Itoh, R Nakashima, R Yaono, S Yoshikawa. 1996. The whole structure of the 13-subunit oxidized cytochrome c oxidase at 2.8 A. Science 272:1136-1144.

van Lis, R, A Atteia, G Mendoza-Hernandez, D Gonzalez-Halphen. 2003. Identification of novel mitochondrial protein components of Chlamydomonas reinhardtii. A proteomic approach. Plant Physiol 132:318-330.

van Lis, R, D Gonzalez-Halphen, A Atteia. 2005. Divergence of the mitochondrial electron transport chains from the green alga Chlamydomonas reinhardtii and its colorless close relative Polytomella sp. Biochim Biophys Acta 1708:23-34.

Weiss, H, HJ Kolb. 1979. Isolation of mitochondrial succinate: ubiquinone reductase, cytochrome c reductase and cytochrome c oxidase from Neurospora crassa using nonionic detergent. Eur J Biochem 99:139-149.
