# Supplementary figures and images for: Parallel Structural Evolution of Mitochondrial Ribosomes and OXPHOS Complexes
Source: Genome Biol Evol. 2015 Apr 9;7(5):1235–51. doi: 10.1093/gbe/evv061 (PMC4453056; doi:10.1093/gbe/evv061)

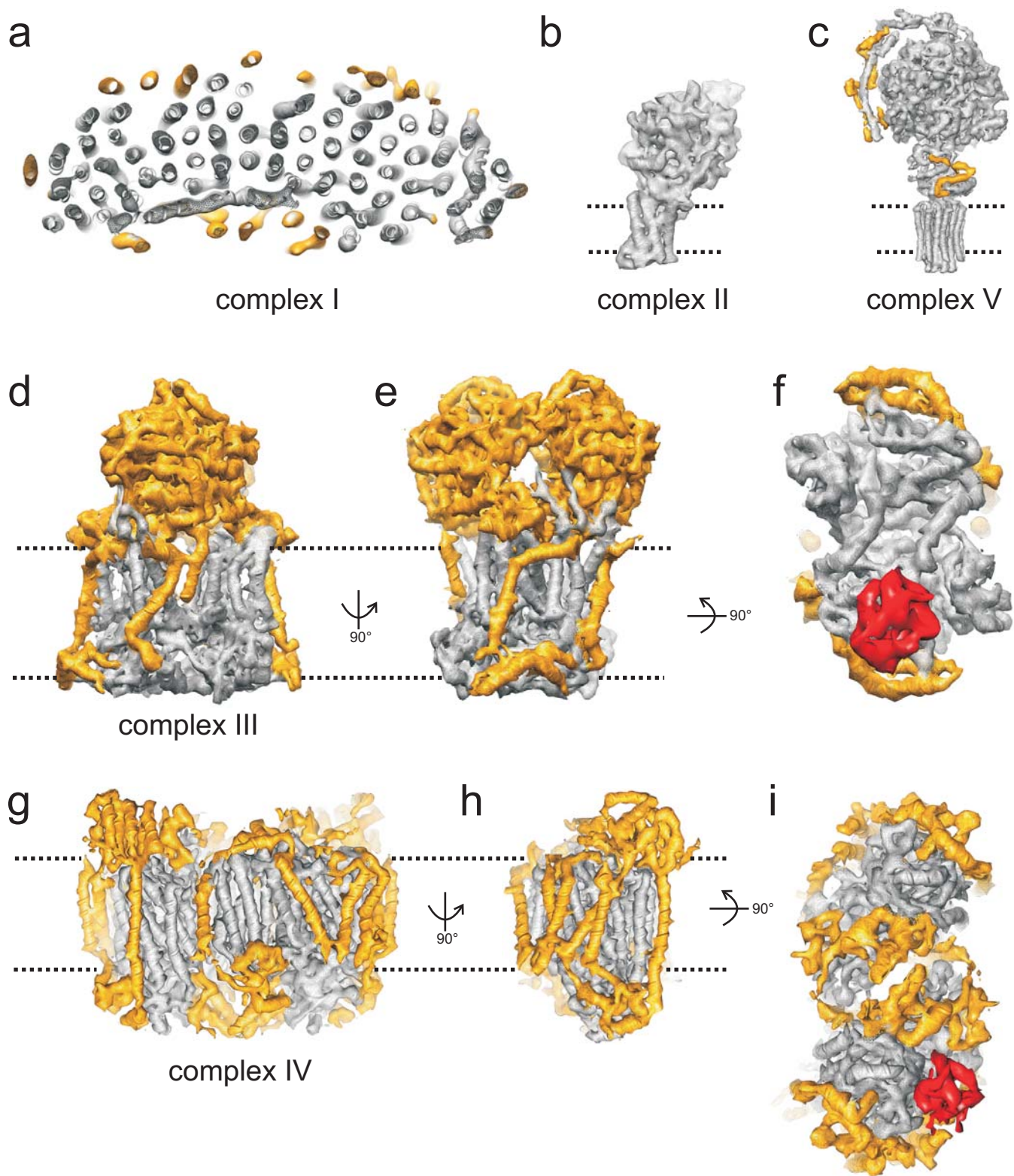

Supplementary Figure 1

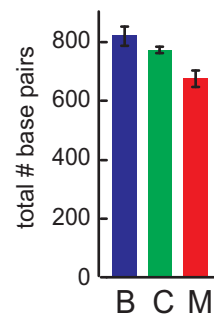

Supplementary Figure 2

Supplement: Supplementary Data [file supp_evv061_Vandersluis_supplementary_fig.pdf]
