# Supplementary material for: Parallel Structural Evolution of Mitochondrial Ribosomes and OXPHOS Complexes
Source: Genome Biol Evol. 2015 Apr 9;7(5):1235–51. doi: 10.1093/gbe/evv061 (PMC4453056; doi:10.1093/gbe/evv061)
Supplement: Supplementary Data [file supp_evv061_Vandersluis_Suppl_Table_I.pdf]

Supplementary Table I

Genome and gene length data from Figure 1 b-g, with organisms listed in Figure 1 a in bold.

Bilateria	mtDNA (kb)	SSU rRNA (nt)	LSU rRNA (nt)	5S rRNA (nt)	total rRNA (nt)	av. tRNA (nt)	cI NAD1-6 (aa)	cIII Cyt*b (aa)*	cIV Cox1-3 (aa)	cV Atp6&8 (aa)
Acipenser dabryanus	16.438	959	1700	-	2659	70.9	2132	380	1008	282
Albinaria caerulea	14.130	759	1035	-	1794	66.0	1959	367	992	269
Alligator mississippiensis	16.646	976	1589	-	2565	72.4	2117	386	1008	278
Ambystoma mexicanum	16.369	927	1554	-	2481	69.5	2109	380	1004	282
Ancylostoma duodenale	13.721	697	958	-	1655	56.0	1838	370	1011	-
Anopheles quadrimaculatus A	15.455	794	1321	-	2115	67.0	2069	378	1003	279
Antrokoreana gracilipes	14.747	799	1284	-	2083	63.9	2032	370	1003	279
Aphonopelma sp. 1 SEM-2008	14.070	691	1062	-	1753	52.7	1930	380	993	270
Asymmetron inferum	15.084	853	1359	-	2212	68.1	2083	380	1007	285
Balaenoptera musculus	16.402	972	1575	-	2547	69.3	2118	379	1004	289
Balanoglossus carnosus	15.708	815	1498	-	2313	71.0	2152	382	1007	281
Biwia zezera	16.599	957	1691	-	2648	70.9	2130	380	1007	281
Bothropolys sp. SP-2004	15.139	726	1183	-	1909	60.7	2006	378	999	271
Branchiostoma lanceolatum	15.076	844	1367	-	2211	66.7	2084	380	1016	281
Bugula neritina	15.433	840	1327	-	2167	62.7	2012	368	1007	270
Caenorhabditis elegans	**13.888**	**513**	**926**	**-**	**1439**	**56.3**	**1841**	**370**	**1011**	**-**
Callinectes sapidus	16.263	785	1323	-	2108	66.6	2051	378	1002	277
Calotes versicolor	16.670	831	1547	-	2378	66.9	2089	374	1000	280
Chelonia mydas	16.497	968	1611	-	2579	70.5	2118	381	1004	288
Ciona intestinalis	14.790	711	1100	-	1811	64.8	2055	361	1011	245
Clymenella torquata	15.538	821	1295	-	2116	64.8	2039	378	1000	284
Diphyllobothrium nihonkaiense	13.747	742	962	-	1704	63.5	1879	368	924	-
Doliolum nationalis	16.351	641	1059	-	1700	63.2	1989	359	1013	250
Drosophila melanogaster	**19.517**	**786**	**1325**	**-**	**2111**	**66.2**	**2060**	**378**	**1001**	**277**
Eptatretus burgeri	17.168	859	1546	-	2405	67.6	2102	385	1007	282
Erpetoichthys calabaricus	16.674	1000	1649	-	2649	70.4	2118	380	1011	269
Eudyptula minor	17.611	975	1608	-	2583	70.4	2122	380	1004	281
Flustrellidra hispida	13.026	653	670	-	1323	55.4	1769	357	988	236
Gallus gallus	16.775	976	1621	-	2597	69.9	2121	380	1003	281
Gymnocrinus richeri	15.966	852	1557	-	2409	70.0	2137	380	1005	285
Heterodontus francisci	16.708	951	1175	-	2126	70.9	2125	381	1009	282
Homo sapiens	**16.571**	**954**	**1558**	**-**	**2512**	**68.7**	**2114**	**380**	**1001**	**294**
Homo sapiens neanderthalensis	16.565	954	1558	-	2512	68.8	2114	378	1000	294
Huso huso	16.760	1013	1700	-	2713	71.1	2132	380	1010	282
Hypochilus thorelli	13.991	688	1041	-	1729	52.2	1937	376	990	271
Lampetra fluviatilis	16.159	902	1615	-	2517	69.3	2108	396	1007	281
Laqueus rubellus	14.017	768	1142	-	1910	58.7	1937	369	994	277
Latimeria chalumnae	16.407	982	1664	-	2646	70.5	2129	380	1006	275
Latimeria menadoensis	16.446	982	1663	-	2645	70.3	2129	380	1005	282
Lepidosiren paradoxa	16.403	929	1577	-	2506	69.9	2116	379	1027	281
Loxocorone allax	14.862	879	1225	-	2104	66.5	2016	378	996	285

Loxosomella aloxiata	15.323	851	1282	-	2133	66.7	2039	378	986	288
Manouria emys	16.455	970	1610	-	2580	70.6	2112	381	1004	281
Myxine glutinosa	18.909	862	1541	-	2403	67.0	2103	385	1007	282
Neoceratodus forsteri	16.572	952	1680	-	2632	70.5	2138	399	1013	282
Octopus vulgaris	15.744	981	1356	-	2337	66.2	2053	379	997	298
Ornithorhynchus anatinus	17.019	942	1561	-	2503	68.6	2107	379	1004	295
Paragonimus westermani	14.965	987	1163	-	2150	64.8	1865	372	924	-
Paraspadella gotoi	11.423	527	936	-	1463	69.0	1834	378	989	-
Patiria pectinifera	16.260	897	1531	-	2428	70.6	2153	379	1006	284
Petromyzon marinus	16.201	900	1621	-	2521	69.6	2108	396	1007	281
Phascolarctos cinereus	16.357	949	1572	-	2521	67.5	2111	379	1001	295
Polypterus ornatipinnis	16.624	950	1655	-	2605	70.2	2116	380	1009	282
Priapulus caudatus	14.919	796	1360	-	2156	65.1	2089	377	1004	279
Python regius	17.245	937	1512	-	2449	65.8	2092	370	1023	281
Rattus norvegicus	16.313	958	1571	-	2529	68.2	2117	380	1002	293
Saccoglossus kowalevskii	17.037	851	1504	-	2355	69.9	2122	382	1007	283
Sardina pilchardus	16.822	952	1681	-	2633	70.6	2157	380	1007	282
Scomber scombrus	16.560	957	1713	-	2670	70.8	2131	380	1010	282
Squalus acanthias	16.738	951	1676	-	2627	70.5	2129	381	1009	282
Tachyglossus aculeatus	16.360	950	1567	-	2517	67.7	2107	379	1004	295
Terebratalia transversa	14.291	762	1105	-	1867	59.8	1988	372	991	279
Tigriopus japonicus	14.628	580	1034	-	1614	62.3	2007	376	1001	260
Vombatus ursinus	16.996	959	1558	-	2517	68.3	2108	381	1002	295
Xenopus laevis	17.553	819	1631	-	2450	69.9	2115	379	1007	281
Xenoturbella bocki	15.234	765	1330	-	2095	66.1	2094	380	1004	286
**Cnidaria, porifera, placozoa**										
Acropora tenuis	18.338	1176	2261	-	3437	70.5	2208	384	1042	304
Agaricia humilis	18.735	1136	1577	-	2713	69.5	2209	383	1035	297
Amphimedon compressa	18.564	1079	2549	-	3628	74.3	2278	382	1026	312
Amphimedon queenslandica	19.960	1324	2872	-	4196	73.3	2270	405	1040	307
Anacropora matthai	17.888	1174	2261	-	3435	70.5	2208	385	1042	304
Aphrocallistes vastus	17.427	918	1718	-	2636	68.0	2287	393	1028	-
Aplysina fulva	19.620	1295	2628	-	3923	73.8	2295	381	1034	321
Aurelia aurita	16.937	960	1817	-	2777	70.5	2256	379	1028	301
Axinella corrugata	25.610	1510	3487	-	4997	73.7	2403	381	1021	334
Briareum asbestinum	18.623	581	2224	-	2805	71.0	2196	380	1041	306
Callyspongia plicifera	18.846	1205	2428	-	3633	74.1	2270	382	1042	326
Chondrilla aff. nucula CHOND	19.282	1194	2469	-	3663	74.3	2300	381	1034	321
Chrysopathes formosa	18.398	1168	2588	-	3756	70.5	2242	380	1028	307
Colpophyllia natans	16.906	1012	1885	-	2897	71.0	2163	379	1008	290
Discosoma sp. CASIZ 168915	20.908	1224	2340	-	3564	70.5	2216	386	1039	301
Discosoma sp. CASIZ 168916	20.912	1068	2342	-	3410	70.5	2216	386	1039	301
Ephydatia muelleri	23.929	1516	2823	-	4339	74.6	2315	381	1046	328
Geodia neptuni	18.020	1244	2389	-	3633	73.5	2285	381	1029	307

Halisarca dujardini	19.277	1188	2490	-	3678	74.3	2310	381	1037	318
Hippospongia lachne	16.755	828	2166	-	2994	70.5	2212	381	1047	306
Igernella notabilis	20.310	1264	3154	-	4418	70.5	2259	381	1014	309
Iotrochota birotulata	19.112	1215	2483	-	3698	73.4	2280	385	1026	316
Keratoisidinae sp. BAL208-1	18.923	1031	1961	-	2992	71.0	2245	386	1046	309
Metridium senile	17.443	1082	2189	-	3271	70.5	2222	381	1040	300
Montastraea annularis	16.138	903	1973	-	2876	70.5	2224	379	1019	290
Montipora cactus	17.887	1172	2337	-	3509	70.5	2208	385	1042	304
Oscarella carmela	20.327	1281	2520	-	3801	73.7	2335	400	1031	311
Pavona clavus	18.315	1169	2299	-	3468	69.5	2212	383	1035	304
Placozoan sp. BZ10101	32.661	1410	3482	-	4892	74.5	2632	383	1097	-
Placozoan sp. BZ2423	36.699	1618	3391	-	5009	73.7	2628	386	1121	-
Placozoan sp. BZ49	37.194	1527	2679	-	4206	74.3	2643	385	1078	-
Plakortis angulospiculatus	19.790	1268	2459	-	3727	71.5	2335	391	1036	313
Pocillopora damicornis	17.415	909	1916	-	2825	70.5	2268	379	1044	295
Porites porites	18.648	1060	2271	-	3331	70.5	2208	386	1034	303
Pseudopterogorgia bipinnata	18.733	924	2211	-	3135	71.0	2173	380	1046	306
Rhodactis sp. CASIZ 171755	20.093	1240	2348	-	3588	70.5	2194	386	1039	301
Ricordea florida	21.376	1218	2447	-	3665	70.5	2214	379	1053	301
Savalia savaglia	20.764	1197	2644	-	3841	71.0	2271	386	1018	304
Seriatopora caliendrum	17.010	916	1902	-	2818	70.3	2181	379	1046	303
Siderastrea radians	19.387	1296	2242	-	3538	70.5	2208	384	1036	309
Suberites domuncula	26.300	1832	3405	-	5237	73.5	2405	383	1042	314
Tethya actinia	19.565	1242	2541	-	3783	73.8	2327	381	1027	324
Topsentia ophiraphidites	19.763	1232	2497	-	3729	73.0	2304	381	1030	311
Trichoplax adhaerens	43.079	1176	2706	-	3882	73.5	2606	383	1088	-
Vaceletia sp. GW948	20.658	821	2511	-	3332	70.5	2242	391	1021	325
Xestospongia muta	18.990	1310	2558	-	3868	74.6	2282	382	1030	320
**Protists, amoebae, non-green algae**										
Acanthamoeba castellanii	41.591	1541	2719	-	4260	74.0	2459	385	-	-
Cafeteria roenbergensis	43.159	1662	2595	-	4257	74.2	2744	379	1073	453
Chondrus crispus	25.836	1376	2583	114	4073	75.3	2407	381	1058	390
Chrysodidymus synuroideus	34.119	1579	2586	-	4165	75.2	2371	389	1069	303
Cyanidioschyzon merolae	32.211	1542	2728	113	4383	75.8	2411	382	1066	385
Desmarestia viridis	39.049	1541	2696	131	4368	75.8	2496	417	-	306
Dictyostelium discoideum	**55.564**	**1555**	**2871**	**-**	**4426**	**74.6**	**2459**	**389**	**-**	**349**
Dictyostelium fasciculatum	54.563	1330	2750	-	4080	74.9	2478	389	-	348
Dictyota dichotoma	31.617	1557	2642	133	4332	75.5	2451	384	1071	336
Emiliania huxleyi	29.013	1548	2688	-	4236	75.8	2379	381	1041	-
Fucus vesiculosus	36.392	1520	2667	134	4321	75.7	2448	418	-	302
Hemiselmis andersenii	60.553	1553	2786	-	4339	75.7	2412	386	1079	370
Laminaria digitata	38.007	1535	2713	130	4378	75.6	2497	390	-	302
Malawimonas jakobiformis	47.328	1573	2731	-	4304	74.9	2383	365	1032	370
Monosiga brevicollis	76.568	1596	2878	-	4474	74.6	2520	380	1053	351

Naegleria gruberi	49.843	1579	2673	-	4252	74.0	2367	497	1211	360
Ochromonas danica	41.035	1563	2591	-	4154	74.2	2413	447	1060	344
Paramecium aurelia	40.469	1681	2568	-	4249	72.5	-	417	-	-
Phytophthora infestans	37.957	1503	2654	-	4157	75.3	2431	383	1055	369
Phytophthora ramorum	39.314	1501	2650	-	4151	75.2	2431	386	1054	369
Phytophthora sojae	42.977	1505	2666	-	4171	75.3	2431	382	1054	369
Polysphondylium pallidum	47.653	1516	2764	-	4280	75.8	2527	386	1197	344
Porphyra purpurea	36.753	1407	2588	-	3995	75.5	2419	382	1071	389
Pylaiella littoralis	58.507	1519	2707	107	4333	75.1	2405	383	-	303
Reclinomonas americana	69.034	1595	2751	110	4456	75.1	2423	390	1058	382
Rhodomonas salina	48.063	1483	2663	-	4146	75.0	2403	388	1075	381
Saprolegnia ferax	46.930	1529	2868	-	4397	75.2	2423	381	1020	364
Tetrahymena malaccensis	47.691	1606	2603	-	4209	75.1	-	426	-	-
Tetrahymena paravorax	47.496	1568	2574	-	4142	74.9	-	426	-	-
Tetrahymena pigmentosa	46.990	1614	2603	-	4217	75.1	-	430	-	-
Tetrahymena pyriformis	47.296	1615	2595	-	4210	74.9	-	430	-	-
Tetrahymena thermophila	**47.577**	**1606**	**2592**	**-**	**4198**	**75.0**	**-**	**426**	**-**	**-**
Thalassiosira pseudonana	43.827	1584	2803	-	4387	76.0	2465	391	1025	332
**Fungi**										
Allomyces macrogynus	57.473	1723	3162	-	4885	76.3	2346	382	1055	309
Ashbya gossypii ATCC 10895	23.564	1570	1680	-	3250	74.1	-	-	-	-
Aspergillus niger	31.103	1445	2833	-	4278	74.4	2496	385	1088	305
Aspergillus tubingensis	33.656	1445	2840	-	4285	73.4	2496	385	1092	305
Beauveria bassiana	29.961	1567	3180	-	4747	74.5	2515	391	1053	311
Candida albicans SC5314	40.420	1462	3131	-	4593	73.8	2167	-	-	294
Candida glabrata	20.063	1650	3093	-	4743	74.4	-	385	1030	308
Candida metapsilosis	24.152	1280	2571	-	3851	73.4	2164	384	1064	294
Candida orthopsilosis	22.528	1324	2799	-	4123	73.4	2186	384	1064	294
Candida parapsilosis	32.745	1325	2680	-	4005	73.3	-	384	1064	294
Candida zemplinina	23.114	1425	2534	-	3959	74.2	-	384	1066	298
Debaryomyces hansonii	29.462	1351	2362	-	3713	74.2	2179	383	1051	294
Epidermophyton floccosum	30.910	1421	2766	-	4187	74.0	-	400	1061	303
Gibberela zeae	42.130	1390	2979	-	4369	74.5	2647	390	1048	314
Hanseniaspora uvarum	18.844	869	1791	-	2660	72.8	-	382	1031	265
Harpochytrium sp. JEL105	24.169	1240	2917	-	4157	73.0	2161	366	989	285
Hyaloraphidium curvatum	29.593	1477	2841	-	4318	73.4	2126	366	998	285
Kluyveromyces lactis	40.291	1738	2928	-	4666	74.3	-	386	1059	304
Kluyveromyces thermotolerans	23.584	1593	3114	-	4707	74.4	-	385	1054	308
Lecanicillium muscarium	24.499	1505	3009	-	4514	74.2	2515	385	1051	315
Metarhizium anisopliae	24.673	1524	3020	-	4544	74.4	2555	390	1044	316
Moniliophthora perniciosa	109.103	1898	4286	-	6184	74.8	2503	396	1058	307
Monoblepharella sp. JEL15	60.432	1387	2830	-	4217	72.4	2145	366	1009	285
Mortierella verticillata	58.745	1491	3300	-	4791	74.7	2491	386	1052	303
Mycosphaerella graminicola	43.964	1673	3370	-	5043	74.1	2796	390	1187	305

Neurospora crassa	**64.840**	**1809**	**3465**	**-**	**5274**	**73.5**	**2685**	**386**	**1079**	**317**
Paracoccidioides brasiliensis	71.335	1418	2625	-	4043	74.3	2439	388	1235	300
Penicillium marneffei	35.438	1381	3066	-	4447	73.9	2501	386	1081	304
Phaesphaeriora nodorum SN15	49.761	689	3263	-	3952	74.0	-	408	-	-
Pichia canadensis	27.694	1537	3009	-	4546	75.0	2521	386	1051	304
Podospora anserina	94.192	1980	3715	-	5695	74.3	2541	388	1060	314
Rhizophydium sp. 136	68.834	1328	2577	-	3905	73.6	2413	370	1033	305
Rhizopus oryzae	54.178	1422	2878	-	4300	74.5	2404	386	1048	307
Schizosaccharomyces japonicu	80.059	2019	4474	-	6493	74.9	-	389	1049	308
Schizosaccharomyces octospor	44.227	1397	2798	-	4195	74.2	-	387	1060	305
Saccharomyces castellii	25.753	1593	3282	-	4875	74.7	-	390	1052	307
Saccharomyces cerevisiae	**85.779**	**1649**	**3295**	**-**	**4944**	**76.6**	**-**	**385**	**1054**	**307**
Saccharomyces servazzii	30.782	1575	2908	-	4483	75.3	-	385	1052	306
Schizophyllum commune	49.704	1863	3576	-	5439	74.8	2997	383	1047	314
Schizosaccharomyces pombe	19.431	1422	2822	-	4244	74.5	-	387	-	305
Smittium culisetae	58.654	1339	2604	-	3943	73.9	2320	396	1049	298
Verticillium dahliae	27.184	1445	2917	-	4362	74.3	2531	390	1058	321
Yarrowia lipolytica	47.916	1591	2971	-	4562	74.6	2353	385	-	303
**Plants & green algae**										
Arabidopsis thaliana	**366.924**	**1935**	**2568**	**118**	**4621**	**78.8**	**2412**	**393**	**1052**	**349**
Beta vulgaris subsp. vulgaris	368.801	1932	3336	119	5387	75.2	2401	393	1049	419
Brassica napus	221.853	1848	3176	119	5143	76.6	2400	393	1052	261
Chaetosphaeridium globosum	56.574	1491	2693	123	4307	75.3	2366	393	1052	428
Chara vulgaris	67.737	1572	2823	116	4511	74.9	2406	383	1040	399
Chlamydomonas eugametos	22.897	1240	1916	-	3156	72.5	-	380	-	-
Chlamydomonas reinhardtii	15.758	1200	2419	-	3619	72.7	-	381	-	-
Chlorokybus atmophyticus	201.763	1713	2947	116	4776	75.6	2495	402	1065	441
Cycas taitungensis	414.903	1837	3037	114	4988	77.5	2396	398	1056	417
Marchantia polymorpha	186.609	1975	2799	122	4896	75.5	2398	404	1038	424
Mesostigma viride	42.424	1558	2843	142	4543	75.8	2407	399	1051	408
Micromonas sp. RCC299	47.425	1458	2303	-	3761	74.7	2477	386	1072	418
Nephroselmis olivacea	45.223	1509	2760	113	4382	75.0	2410	391	1041	415
Nicotiana tabacum	430.597	1902	3406	119	5427	75.2	2423	393	1052	-
Oltmannsiellopsis viridis	56.761	1539	2669	109	4317	74.9	2451	426	1049	414
Oryza sativa Indica Group	491.515	1695	3542	122	5359	73.0	2438	397	1064	-
Ostreococcus tauri	44.237	1472	2586	-	4058	75.8	2455	399	1050	415
Pedinomonas minor	25.137	1178	2110	-	3288	75.7	2263	374	-	295
Physcomitrella patens	105.340	1587	2946	122	4655	76.1	2407	406	1023	426
Polytomella capuana	12.998	1028	1566	-	2594	73.0	-	369	-	-
Prototheca wickerhamii	55.328	1674	3009	117	4800	75.3	2474	384	1035	483
Pseudendoclonium akinetum	95.880	1424	2776	-	4200	76.0	2534	415	1063	403
Scenedesmus obliquus	42.781	1634	2410	-	4044	75.4	2454	399	995	-
Sorghum bicolor	468.628	1961	3531	118	5610	75.1	2467	388	1055	537
Tripsacum dactyloides	704.100	1964	3552	126	5642	75.2	2529	388	1235	562

Triticum aesitvum	452.528	1955	3467	122	5544	75.2	2443	398	1049	542
Zea luxurians	539.368	1951	3542	126	5619	75.4	2571	388	1053	563
Zea mays subsp. mays	569.630	1968	3552	126	5646	74.6	2416	388	1053	563
Zea mays subsp. parviglumis	680.603	1968	3552	126	5646	75.4	2416	388	1053	563
Zea perennis	570.354	1958	3562	126	5646	75.4	2405	388	1053	563
**chloroplast *Spinacea olerace***	**150.725**	**1491**	**2810**	**121**	**4422**	**75.4**	**2515**	-	-	**433**
