# Supplementary material for: Parallel Structural Evolution of Mitochondrial Ribosomes and OXPHOS Complexes
Source: Genome Biol Evol. 2015 Apr 9;7(5):1235–51. doi: 10.1093/gbe/evv061 (PMC4453056; doi:10.1093/gbe/evv061)
Supplement: Supplementary Data [file supp_evv061_Vandersluis_Suppl_Table_IV.pdf]

Supplementary Table IV

Genbank accession numbers of MS-OPs from OXPHOS complexes III and IV

Rsp: Rhodobacter sphaeroides; Ddi: Dictyostelium discoideum; Sce: Saccharomyces cerevisiae; Dme: Drosophila melanogaster;
Hsa: Homo sapiens; Bta: Bos taurus; Cel: Caenorhabditis elegans; Tth: Tetrahymena thermophila; Ngr: Naegleria gruberi; Ath: Arabidopsis thaliana

	**Complex III**	***Rsp***	***Ddi***	***Sce***	***Dme***	***Hsa***	***Bta***	***Cel***	***Tth***	***Ngr***	***Ath***
KOG3052	Cytc1	116668439	66801175	117766	7295470 7301764	21359867	109940069	17506225	118389517	290976633	15237497 15232125
KOG4663	Cytb	114793883	7524981	6226529	5835245	251831119	117843	5834888	156511197	11466189	27808646
KOG1671	ISP/SU 9	114793885	166240368	136715	33636485	143811471	1351360	74958505	146164447	290995600	15240627
KOG0960	Core 1	-	111226358	51013847 51013545	7299936	46593007	10720406	17553678	146181190	290986442	15232845
KOG2583	Core 2	-	66803202	45270026	7294089	50592988	401248	17555260 17570205 17569737	229594165	290973053	15218090 18401141
KOG3440	SU 6	-	66808287	1717954	7293193 7301665	5454152	109940044	17535957	-	290980151	15236811 15239430
KOG4116	SU 7	-	66813752	45270998	7293979	83367083	1351362	17569317 17534121	-	-	15239099
KOG4763	SU 8	-	111226950	1174866	281366757	83627705	109940045	17555308	146145137	-	238478492 15226177
KOG3494	SU 10	-	66810490	136702	7303402	41281885	109940043	297206945	-	-	15231675
-	SU 11	-	-	586146	24654465	5803217	3915912	25151097	-	-	-
											18405497

	**Complex IV**	***Rsp***	***Ddi***	***Sce***	***Dme***	***Hsa***	***Bta***	***Cel***	***Tth***	***Ngr***	***Ath***
KOG4769	Subunit I	21955871	2251097	6226519	5835235	251831109	60101827	5834891	15027668	11466182	13449404
KOG4767	Subunit II	15022167	2251097	6226540	5835236	251831110	60101828	5834892	15027663	11466213	13449304
KOG4664	Subunit III	75345336	7524973	6226542	5835239	251831113	60101831	5834889	-	11466186	26556997
	CoxIV	22417150	-	-	-	-	-	-	-	-	-
KOG4075	Subunit IV	-	66812344	6322080 6324276	7298641 21626865	4502981 17999526	506389	17509657	-	-	-
KOG4077	Subunit Va	-	-	6321842	7299519	190885499	42564216	17555666	-	-	-
KOG3352	Subunit Vb	-	66826157	3582	3876417 7297136	180941	74268063	3876417	146165583	290988984	21592567
KOG3469	Subunit VIa	-	-	6321247	7291601 23093179	17999528 4885149	404387	17553574	-	-	15235720
KOG3057	Subunit VIb	-	60467075	6323067	22832604	4502985 17467773	28603814	17556420	-	-	15219886 15223279 15234375
-	Subunit VIc	-	-	171299	22945660	3859868	117306607	71981252	-	-	-
-	Subunit VIIa	-	-	3585	24645039	3041844	162895	-	-	-	-
-	Subunit VIIb	-	-	-	-	4502991	162959	-	-	-	-
KOG4527	Subunit VIIc	-	-	6323427	21645512	4502993	6015488	25143040	-	-	-
-	Subunit VIII	-	-	-	-	4758044	162899	-	-	-	-
											2275216 222423706 88010838
											15241035
											15236960
											18409974
											18415628
											15229280
											20198032
